# Metabolomics-derived marker metabolites to characterize *Phaeocystis pouchetii* physiology in natural plankton communities

**DOI:** 10.1038/s41598-020-77169-w

**Published:** 2020-11-24

**Authors:** Constanze Kuhlisch, Julia Althammer, Andrey F. Sazhin, Hans H. Jakobsen, Jens C. Nejstgaard, Georg Pohnert

**Affiliations:** 1 Institute for Inorganic and Analytical Chemistry, Friedrich Schiller University Jena, Lessingstraße 8, 07743 Jena, Germany; 2 Shirshov Institute of Oceanology, Russian Academy of Sciences, Nakhimovsky Prospect 36, Moscow, Russia; 3 Department of Bioscience, Aarhus University, Frederiksborgvej 399, 4000 Roskilde, Denmark; 4 Leibniz-Institute of Freshwater Ecology and Inland Fisheries, Dep. 3, Alte Fischerhütte 2, 16775 Stechlin, Germany; 5 Present Address: Department of Plant and Environmental Sciences, Weizmann Institute of Science, 234 Herzl Street, 7610001 Rehovot, Israel; 6 Present Address: JenaBios GmbH, Löbstedter Straße 80, 07749 Jena, Germany

**Keywords:** Microbial communities, Microbial ecology, Chemical ecology, Metabolomics

## Abstract

*Phaeocystis pouchetii* (Hariot) Lagerheim, 1893 regularly dominates phytoplankton blooms in higher latitudes spanning from the English Channel to the Arctic. Through zooplankton grazing and microbial activity, it is considered to be a key resource for the entire marine food web, but the actual relevance of biomass transfer to higher trophic levels is still under discussion. Cell physiology and algal nutritional state are suggested to be major factors controlling the observed variability in zooplankton grazing. However, no data have so far yielded insights into the metabolic state of *Phaeocystis* populations that would allow testing this hypothesis. Therefore, endometabolic markers of different growth phases were determined in laboratory batch cultures using comparative metabolomics and quantified in different phytoplankton blooms in the field. Metabolites, produced during exponential, early and late stationary growth of *P. pouchetii*, were profiled using gas chromatography-mass spectrometry. Then, metabolites were characterized that correlate with the growth phases using multivariate statistical analysis. Free amino acids characterized the exponential growth, whereas the early stationary phase was correlated with sugar alcohols, mono- and disaccharides. In the late stationary phase, free fatty acids, sterols and terpenes increased. These marker metabolites were then traced in *Phaeocystis* blooms during a cruise in the Barents Sea and North Norwegian fjords. About 50 endometabolites of *P. pouchetii* were detected in natural phytoplankton communities. Mannitol, *scyllo*-inositol, 24-methylcholesta-5,22-dien-3β-ol, and several free fatty acids were characteristic for *Phaeocystis*-dominated blooms but showed variability between them. Distinct metabolic profiles were detected in the nutrient-depleted community in the inner Porsangerfjord (< 0.5 µM NO_3_^−^, < 0.1 µM PO_4_^3−^), with high relative amounts of free mono- and disaccharides indicative for a limited culture. This study thereby shows how the variable physiology of phytoplankton can alter the metabolic landscape of entire plankton communities.

## Introduction

The marine microalga *Phaeocystis pouchetii* (Hariot) Lagerheim, 1893 is a key phytoplankton species in the Arctic and high latitude areas, especially along the Norwegian coast and in the Barents Sea^1^. Several single-celled stages are known or hypothesized within its life cycle, however, most spring blooms are characterized by the colony stage of the alga^2–4^. Colonies develop from dividing single cells excreting a polysaccharide-rich mucus that builds the matrix for associations of several hundreds of cells^5^. These carbohydrate-rich particles contribute to the microbial loop and vertical flux^6,7^, as well as the marine pelagic food web. The palatability and intake of *Phaeocystis* by grazers, its nutritional value, and chemical defence strategies have been investigated intensively for decades^8^. *P. pouchetii* colonies are ingested by various grazers including dinoflagellates, ciliates, copepods, amphipods, and fish. Other grazers avoid colonies or select specific colony size ranges. Size selection mechanisms, as well as algal bloom phase-dependent biochemical mediation, have been suggested^9,10^. Recent modelling of the Arctic marine ecosystem under climate warming predicted a decrease in sea ice coverage and an increase in gross primary production^11^. *P. pouchetii* may expand its distribution and occurrence as already observed locally^12,13^. Within this context, the relevance of *P. pouchetii* biomass for the trophic transfer in the Arctic food web needs further investigation.

The role of *P. pouchetii* within the Arctic food web has been investigated under laboratory, semi-natural, and natural conditions with contradicting results^8^. Whereas in some experiments both single cells and colonies are ingested by zooplankton grazers^14^, in others only colonies were grazed^15^, or the copepod grazers avoided healthy colonies ingesting only senescent ones^9^. Senescent colonies of *P. pouchetii* blooms around Spitsbergen and the Barents Sea were actively grazed by mesozooplankton, however, not by microzooplankton^16,17^. Whereas microzooplankton actively grazed upon single cells of *P. pouchetii* during blooms in mesocosm experiments, the colonies were not efficiently ingested neither by micro- nor mesozooplankton^18,19^. In contrast, high microzooplankton grazing on single cells and small colonies of *P. pouchetii* may have even inhibited bloom-formation in a North Norwegian fjord^20^. In conclusion, zooplankton grazing may rely both on *Phaeocystis* colony formation and bloom phase with a possible underlying modulating effect of the metabolite composition of the cells.

Indeed, phytoplankton cells do not represent steady-state chemical compartments. Cell metabolism is highly plastic responding to both biotic and abiotic environmental conditions. As shown for laboratory batch cultures of the diatoms *Skeletonema marinoi* and *Thalassiosira pseudonana*, the haptophyte *Emiliania huxleyi*, and the cyanobacterium *Synechococcus*, the composition and release of metabolites from phytoplankton cells is growth phase-dependent^21–25^. The responsiveness of herbivores to algal metabolite composition can substantially influence grazing rates, and thereby shape predator–prey interactions^22^. Algal fatty acid profiles can further alter the chemical composition of predator cues that mediate diverse grazing defence strategies in their algal prey^26^. Metabolic variability in algal endo- and exometabolomes is also expected in natural phytoplankton communities, but was never demonstrated, and could be a determining factor for the grazing on *P. pouchetii*.

In metametabolomics approaches, complex natural communities are investigated in contrast to defined monoclonal laboratory cultures. Untargeted metabolite profiling of marine phytoplankton communities has been applied to search for novel metabolites^27,28^, characterize marine particulate organic matter^29,30^, support metabarcoding studies^31^, and screen for sulphur compounds^32^. However, whereas algal endometabolomes are frequently investigated under laboratory conditions, data on phytoplankton bloom metabolomes is scarce. This study aims to define intracellular marker metabolites for growth phases of *Phaeocystis pouchetii* laboratory cultures using comparative metabolomics^33^. With these marker metabolites, natural phytoplankton communities were analysed and their bloom physiology characterized in a metametabolomics approach. Parts of this study are taken from the PhD Thesis by C. Kuhlisch^34^.

## Results

### Growth of *Phaeocystis pouchetii* AJ01 in batch culture

**Figure 1:**
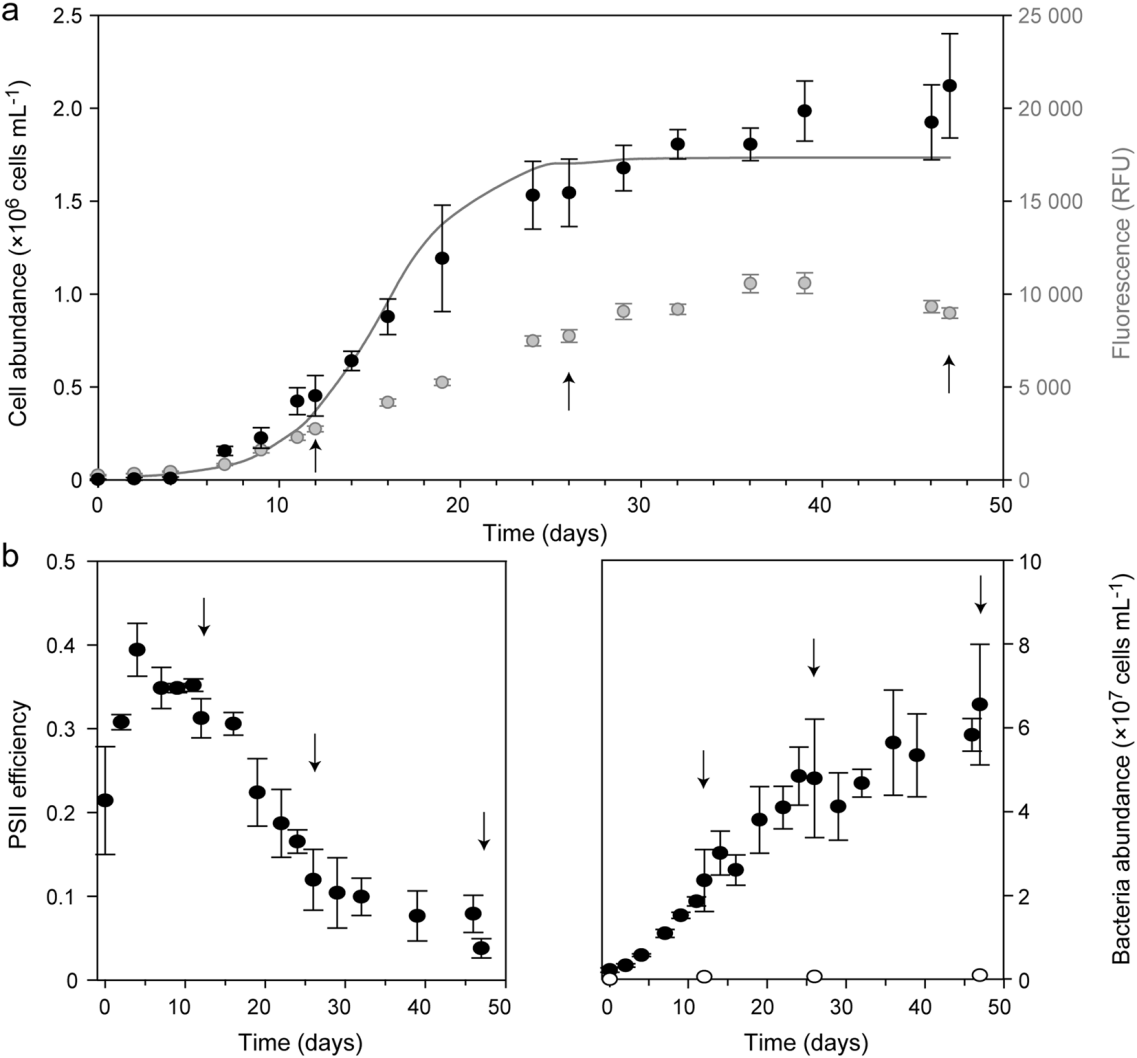
*Phaeocystis pouchetii* AJ01 growth-associated parameters as average ± standard deviation (n = 4). (**a**) Algal abundance (cells mL^−1^; black), Chl *a* fluorescence (RFU = relative fluorescence units; grey), and fitted logistic growth model (solid line; 3.7 × 10^5^ deviance). (**b**) Algal PSII efficiency (left panel) and bacterial abundance (cells mL^−1^; right panel) within the cultures (filled circles) and the control (open circles). Arrows indicate sampling days of endometabolites.

The metabolic plasticity of *P. pouchetii* was investigated by comparing endometabolomes at different growth phases. A strain with just one morphotype was selected to prevent potential overlaying effects of morphotype heterogeneity. Batch cultures were set up, monitored every 2–3 days throughout 47 days, and sampled three times for endometabolite profiling. All cultures showed logistic growth (Fig. 1a). Light microscopy confirmed one flagellated single cell type during the whole growth period. This is in accordance with the reported synchronized cell divisions taking place during the dark phase^35^. Directly after inoculation, the cultures remained in lag-phase for 4 days showing no significant changes in cell abundance or Chl *a* fluorescence (Fig. 1a). In parallel, photosystem II (PSII) efficiency increased, indicating the recovery of the cells after transfer to fresh medium (Fig. 1b, left panel). During the following 12 days, cell numbers increased significantly between days (t-test, α = 0.05) with an average specific growth rate of µ = 0.38 ± 0.04 day^−1^, whereas PSII efficiency remained at 0.31–0.35 ± 0.02. Metabolites were sampled on day 12. During the following 20 days, cell abundances levelled off at about 2 × 10^6^ cells mL^−1^ indicating the stationary growth phase. Chl *a* fluorescence slightly increased further, whereas PSII efficiency first decreased to 0.1 ± 0.04 on day 29 (= early stationary growth, sampled on day 26), and then remained low until day 46. During the last two days, Chl *a* fluorescence decreased significantly (t-test, α = 0.05) and PSII efficiency dropped to 0.04 ± 0.01, while no significant changes in cell abundance occurred (= late stationary growth, sampled on day 47).

Nutrient concentrations can explain this growth development (Supplementary Fig. S1). During lag and exponential growth, neither nitrate nor phosphate was limiting, nitrite was below the detection limit. Nitrate decreased from about 800 µM to 600 µM at the end of the experiment, thus cultures were not N-limited. Nitrite increased from day 22 onwards to 4.5 µM, which may indicate light limitation, as low irradiance levels can restrict cellular nitrite reduction leading to nitrite excretion^36^. This may have induced the stationary growth phase. Phosphate fell below 1 µM on day 36 and may have co-limited algal growth during the last two weeks of the experiment.

Algal cultures showed an initial background of 2 × 10^6^ bacteria mL^−1^, increasing exponentially during algal exponential growth, and levelling off around 5 × 10^7^ bacteria mL^−1^ (Fig. 1b, right panel). Bacterial metabolites may contribute to the sampled metabolite profiles, however, with an average carbon content of 20 fg carbon per cell for bacteria^37^ and 10^7^ pg carbon per cell for *P. pouchetii*^38^, bacteria accounted for less than 0.1% of the cellular carbon content at the end of the experiment. Co-occurring bacteria can further affect algal growth and metabolism^39–41^. However, as also the absence of bacteria affects algal growth and some microalgae cannot be cultured under axenic conditions^42,43^, the *P. pouchetii* cultures were not made axenic before experimental setup.

### Growth phase-specific endometabolites of *P. pouchetii* cultures

**Figure 2:**
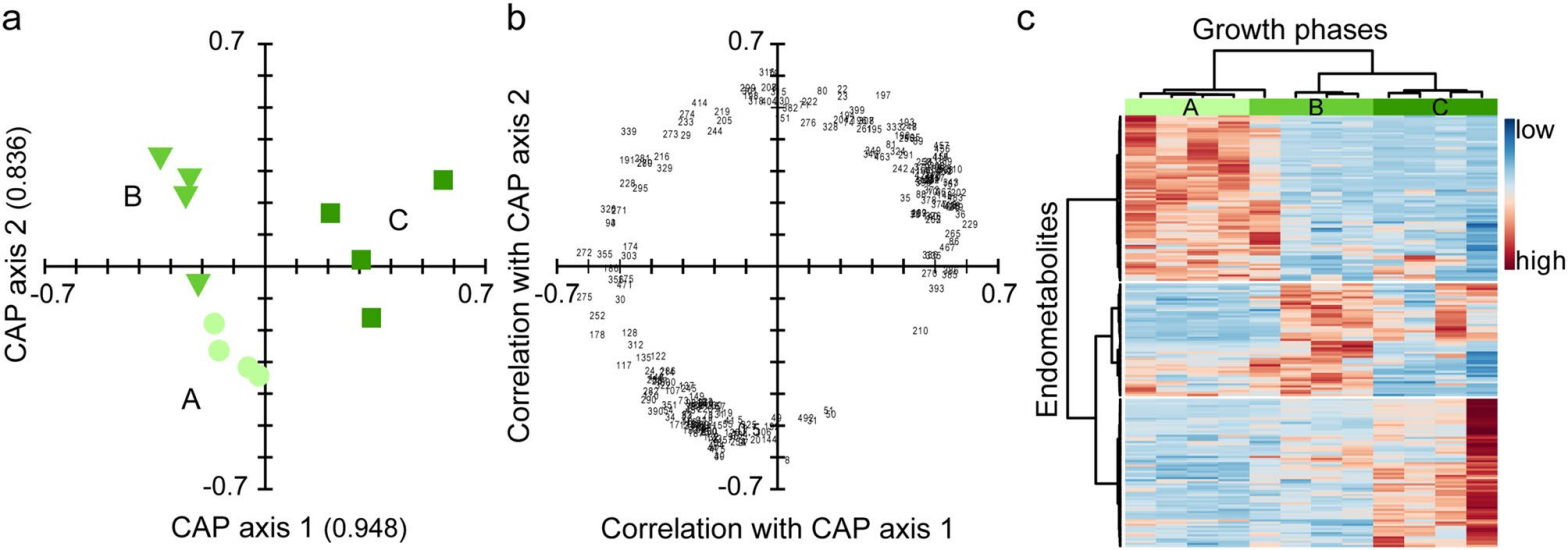
Endometabolic changes of *Phaeocystis pouchetii* AJ01 during growth in laboratory batch cultures (n = 4). Separation is based on canonical analysis of principal coordinates (CAP); axis eigenvalues are given in parentheses. (**a**) Score plot of the growth phases: exponential phase ('exp', bright green circle), early stationary phase ('e.stat', green triangle), late stationary phase ('l.stat', dark green square). (**b**) Loading plot (scaled to score plot) of correlated endometabolites (> 0.708) depicted with their identifier as tabulated in the Supplementary Table S6. (**c**) Heat map of correlated endometabolites (analyte peak area/peak sum) indicating low (blue) or high (red) relative concentrations in the growth phases. Metabolites and samples are clustered hierarchically for better visualization using the Pearson distance measure and Ward cluster algorithm.

Metabolic pathways are undergoing substantial quantitative and qualitative changes during growth. In batch cultures, physiological adaptations occur simultaneously with the developing growth environment. Overall, 518 features were detected by AMDIS and integrated by MET-IDEA. After data pre-processing, 477 features remained for data analysis: 458 ± 6 from exponential growth, 431 ± 27 from early, and 398 ± 57 from late stationary growth (n = 4). Multivariate statistical analysis of the sum normalized peak areas allowed to differentiate the three growth phases. Initially, an unsupervised principal coordinate analysis (PCO) was conducted, with the first two axes explaining 78% sample variation. Using the PCO axes, a supervised canonical analysis of principal coordinates (CAP) was conducted (Fig. 2a). The replicates of each growth phase grouped together, whereas the growth phases were separated by two axes (eigenvalues: axis 1 = 0.948, axis 2 = 0.836; squared canonical correlations: axis 1 = 0.898, axis 2 = 0.698). Separation of the growth phases was confirmed by cross validation (1 out of 12 samples misclassified) and permutation test (n = 9999, p < 0.001 for trace statistic). Based on a critical value of 0.708 (test of multiple correlation using t; n = 12), 225 metabolites were significantly correlated with the CAP axes (Fig. 2b, Supplementary Table S6) from which 107 were highly correlated (> 0.8). After removal of features with a retention time < 5 min and artefacts (doublets, signal-to-noise ratio < 10, contaminants), 88 features remained for identification. From those, 31% were identified (n = 27, in the following mentioned by their name), 3% putatively annotated (n = 3, in the following mentioned as putative), 41% putatively assigned to metabolite classes (n = 36, in the following discussed as classes), and 25% remained unknown (n = 22). Most metabolites belonged to the class of saccharides, sugar alcohols, and sugar acids (n = 31), followed by fatty acids and derivatives (n = 11), and amino acids (n = 9). Many metabolites had their highest relative concentration in either the exponential phase (n = 32; e.g. amino acids) or the late stationary phase (n = 33; e.g. sterols), whereas only 12 metabolites were correlated with the early stationary phase (e.g. glycerol and *scyllo*-inositol; Fig. 2c).

Saccharides, sugar acids and sugar alcohols were the most pronounced regulated substance classes showing strong interphase variability. Most saccharides were significantly correlated with the late stationary phase with either constantly increasing relative concentrations (e.g. xylose, ribose, maltose), or strong up-regulation in both stationary phases (e.g. glyceraldehyde). On the contrary, most sugar acids (e.g. threonic acid) were significantly correlated with the exponential phase with decreasing relative concentrations towards the late stationary phase. Most of the sugar alcohols (e.g. glycerol, *scyllo*-inositol) were correlated with the early stationary phase, whereas one unidentified sugar alcohol (met. 275) was up-regulated in the late stationary phase. In this phase, mannitol showed strong down-regulation, as did two pentafuranoses (met. 186, 194) and one sugar acid (putatively identified as quinic acid, Supplementary Fig. S2). Fatty acids and derivatives—the second most pronounced regulated substance class—were primarily correlated with late stationary growth (e.g. arachidonic acid (AA), eicosapentaenoic acid (EPA), 1-palmitoyl-glycerol), or both the early and late stationary phase (putative octadecadienoic- and octadecatrienoic acid). The only exceptions were tetra- and hexadecanoic acid, which showed higher relative concentrations in the exponential (tetra-) and early stationary phase (hexa-). Amino acids (e.g. glycine, alanine, serine, valine, threonine) were correlated with the exponential phase and decreased towards the late stationary phase. Representatives of other metabolite classes were either correlated with the exponential phase (e.g. pyrrole-2-carboxylic acid), or with the late stationary phase (e.g. α-tocopherol, an unidentified alcohol (met. 155), three hydrocarbons (putative dodecatriene, met. 389, met. 433), and two sterols (24-methylcholesta-5,22-dien-3β-ol, met. 452)).

### Blooms of *Phaeocystis pouchetii* in natural phytoplankton communities

**Figure 3:**
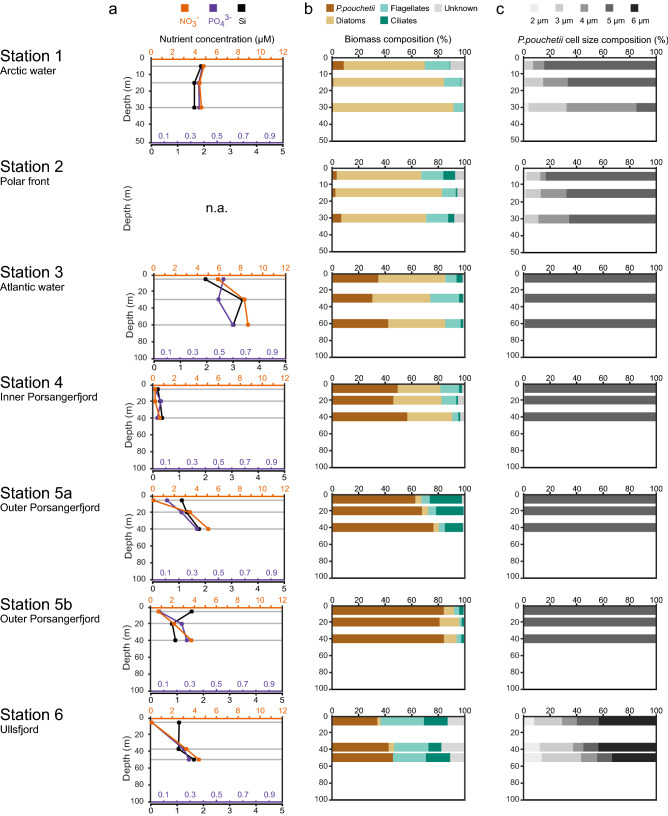
PHAEONIGMA cruise metadata of stations sampled along a transect in the Barents Sea (Station 1–3) and in North Norwegian fjords (Station 4–6). The geographic location of the cruise station is given in Fig. 5a. (**a**) Depth profiles of dissolved inorganic nutrient concentrations (µmol L^−1^) of nitrate (orange), phosphate (violet), and silicate (black). (**b**) Biomass proportion (% mg C m^−3^) of phytoplankton taxa classified as '*Phaeocystis pouchetii*', 'diatoms', 'flagellates', 'ciliates', and 'unknown'. (**c**) Biomass proportion (% mg C m^−3^) of different cell sizes of *P. pouchetii* ranging from 2 µm (light grey) to 6 µm (dark grey). Phytoplankton community characteristics were determined by microscopy (for absolute numbers see Supplementary Table S7). Grey horizontal lines represent the sampling depths above, at and below the Chl *a* maximum for metabolite profiling. *n.a.* not available.

Several phytoplankton communities were sampled along a transect in the Barents Sea (Stations 1–3) and along the North Norwegian coast (Stations 4–6) capturing different bloom scenarios (Fig. 3, Supplementary Figs. S3, S4). Station 1 was located at the ice edge in Arctic waters; slight stratification and non-limiting nutrients allowed a biomass-rich diatom bloom dominated by *Gyrosigma tenuirostrum*, *Thalassiosira* spp., and *Odontella aurita*. Station 2 was located in the polar front region, where a well-mixed water column allowed a diatom bloom with low biomass (*Thalassiosira* spp., *O. aurita*, *Fragilariopsis oceanica*). Station 3 was located within North Atlantic waters characterized by an elevated temperature and salinity, a well-stratified water column, and high nutrient concentrations. The phytoplankton community was dominated in biomass by diatoms (*Thalassiosira* spp., *Skeletonema costatum*, *Chaetoceros* spp.), with a high abundance of *P. pouchetii* colonies (2.7 × 10^3^ cells/mL at Chl *a* max.). Within the colonies, individual cells were distributed in well-organized patches, and a few colonies showed infestation by the pennate diatom *Pseudo-nitzschia* spp. sensu (Fig. S4a) indicating an early stage of the *Phaeocystis* bloom. Station 4 was located in the inner Porsangerfjord and was characterized by limiting nutrient concentrations (Chl *a* max.: nitrate below detection level, 0.05 µM phosphate, 0.22 µM silicate). The algal bloom was dominated in biomass by diatoms (*Thalassiosira* spp., *Thalassiosira nordenskioldii*, *Chaetoceros socialis*), with a high abundance of *P. pouchetii* colonies (1.8 × 10^4^ cells/mL at Chl *a* max.). The cell patches within colonies were slightly denser compared to Station 3, and *Pseudo-nitzschia* spp. was present in only a few occasions (Fig. S4b). Station 5a and 5b were both located in the outer Porsangerfjord with a shallow mixed upper water layer and Chl *a* maximum (0–20 m), and with low nutrient concentrations (< 0.7 µM nitrate, < 0.12 µM phosphate). The phytoplankton bloom was dominated by *P. pouchetii* with a high abundance of colonies (up to 75% biomass and 3.3 × 10^4^ cells/mL). Colony infestation with *Pseudo-nitzschia* spp. was a prominent feature at both Station 5a and 5b, whereas at Station 5b there was also evidence of *Phaeocystis* cells leaving the organized patch structure, forming flagellated cells that were spread more-or-less evenly in the colony matrix (Fig. S4c-d). Station 6 was located in the outer Ullsfjord characterized by a deep phytoplankton bloom (Chl *a* max.: about 40 m). The phytoplankton community comprised ciliates, flagellates and *P. pouchetii* (40% biomass contribution at 50 m). In contrast to previous stations, the abundance of *Phaeocystis* colonies increased with depth (6.4 × 10^3^ cells/mL at 50 m), and cell sizes varied between 2–6 µm with a high proportion of large cells (6 µm: up to 47%). Furthermore, progressed colony breakdown was apparent by cells that had left the organized patch structure and were evenly spread in the colonies (Fig. S4e).

In summary, *P. pouchetii* characterized the phytoplankton communities at Stations 3–6 with high colony abundances and clear signs of bloom stage progression along the station transect. These stations are therefore well suited to test, if growth phase-specific endometabolites as determined from *P. pouchetii* laboratory cultures can be detected in the field and used to characterize the physiology of natural *Phaeocystis* blooms.

### Metabolic landscape of natural plankton communities

**Figure 4:**
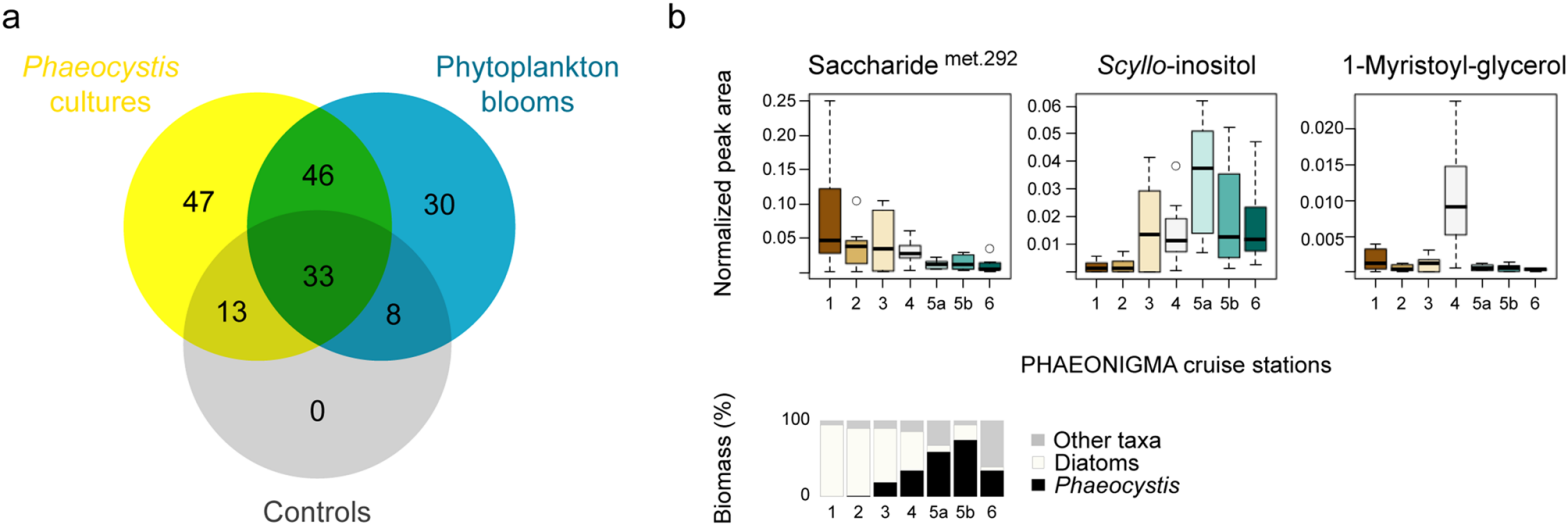
Occurrence of *Phaeocystis pouchetii* endometabolites within natural phytoplankton communities of the Barents Sea and North Norwegian fjords. (**a**) Venn diagram of unique and shared metabolites between *P. pouchetii* laboratory cultures (n = 12), natural phytoplankton communities (n = 58), and controls (n = 7). (**b**) Box-Whisker plots showing abundance of selected shared metabolites (as peak area/peak sum) at Barents Sea (1–3) and North Norwegian fjord stations (4–6; n = 8–9, averaged over all depths). The geographic locations of the cruise stations are depicted in Fig. 5a. The depicted metabolites are representative for three occurrence patterns: diatom dominance (station 1–3), *Phaeocystis* dominance (station 5–6), and nutrient depletion (station 4) as indicated by the relative biomass of phytoplankton taxa at the respective sites.

In total, 572 features were extracted from all laboratory and field samples. After removal of features that occurred in low abundance, not in triplicates, not within the range of the retention time standards, or belonged to internal standards, 182 features remained. Manual curation revealed that 30% of the remaining features were detected in at least one control sample (Fig. 4a), and were omitted from further discussion. Thus, 47 features were unique for *Phaeocystis* cultures, 30 features were unique for field samples, and 46 features were shared between laboratory and field samples (Fig. 4a, Supplementary Table S8). Most of the shared features (~ 70%) showed growth phase-dependence in cultures. Hierarchical cluster analysis of normalized peak areas averaged per station (Supplementary Fig. S5) revealed three main occurrence patterns in the field: high abundance at diatom-dominated stations, at *P. pouchetii*-dominated stations, or at the nutrient-depleted station 4 (Fig. 4b, Supplementary Table S8). Within the metabolites abundant at *P. pouchetii*-dominated stations were the sugar alcohols mannitol and *scyllo*-inositol (Fig. 4b), the fatty acids octadecatetraenoic acid and docosahexaenoic acid, 1-stearoyl-glycerol, and the sterol 24-methylcholesta-5,22-dien-3β-ol. The fatty acids hexadecanoic acid, octadecanoic acid, octadecenoic acid, and several unknown metabolites formed a subclade with higher abundance at station 3 and lower at station 6. The nutrient-depleted station 4 was characterized by high abundances of mono- and disaccharides (ribose, maltose, several unknown saccharides), fatty acids and derived metabolites (hexadecenoic acid, 1-myristoyl-glycerol (Fig. 4b), 1-palmitoyl-glycerol), and phytol. Within the metabolites abundant at diatom-dominated stations were threonic acid, several saccharides (e.g. erythrose and met. 292 (Fig. 4b)), fatty acids such as EPA, and three phytol-like structures. Thus, several of the laboratory-derived endometabolites were re-detected in natural phytoplankton blooms. While some were indeed associated with *Phaeocystis* biomass, some showed stronger association with other taxa like diatoms (Supplementary Table S4). Within the *Phaeocystis* blooms, several *Phaeocystis* endometabolites varied strongly independent of biomass, thereby indicating additional environmental constraints such as nutrient availability (Supplementary Table S5).

**Figure 5:**
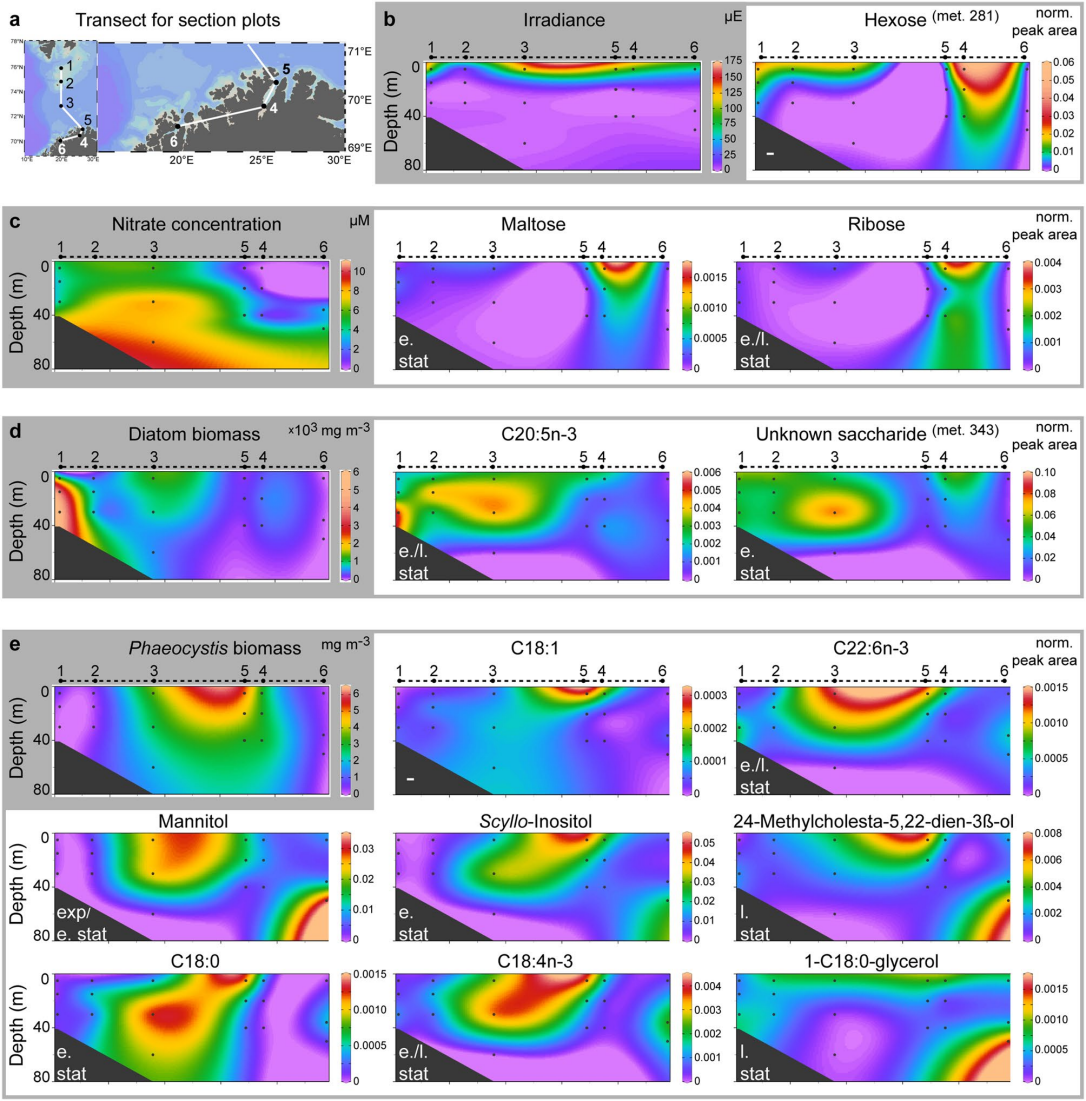
Relative abundance of *P. pouchetii* endometabolites within phytoplankton communities in the Barents Sea and North Norwegian fjords. (**a**) Transect along the PHAEONIGMA cruise stations 1–6 as used for section plots. (**b**–**e**) Section plots of metabolites that can be linked to species composition and environmental parameters (shaded in grey). Metabolites with similar occurrence patterns are grouped in grey boxes. 'Met.'—metabolite identifier within laboratory dataset. The growth phase of highest abundance for each metabolite is indicated (exp—exponential, e.stat—early stationary, l.stat—late stationary). The station map and section plots were generated using Ocean Data View (version 4.7.10, https://odv.awi.de).

## Discussion

### Growth phase-specific endometabolites of *P. pouchetii*

It has long been supposed that the metabolic plasticity of phytoplankton may influence zooplankton grazing and transfer rates, especially for the haptophyte *Phaeocystis*^8^. Until recently, however, thorough metabolite analyses of the prey have not been included in grazing studies. When included, it has indeed been shown that phytoplankton species, such as the diatom *Skeletonema marinoi*, can change during growth from an avoided to a highly ingested food source in accordance with changes in their endometabolome^22^. Endometabolic plasticity during growth has been demonstrated for rather few microalgae, namely *S. marinoi* and *E. huxleyii*^23,24^. This study on *P. pouchetii* is the first report of endometabolic plasticity for this dominant Arctic and Boreal haptophyte. Physiological changes in batch cultures of *P. pouchetii* were characterized using untargeted metabolomics. Growth phases as determined by cell abundance and Chl *a* fluorescence were associated with distinct endometabolic states with pronounced alternations in amino acid, fatty acid, and carbohydrate metabolism.

Exponential growth was characterized by free amino acids reflecting high metabolic activity towards proteins under nitrogen-replete conditions. Increased levels in free amino acids during intense growth were previously reported for diatoms and higher plants^44^. The observed elevated levels in glutamate may be directly linked to nitrogen incorporation, whereas serine and glycine are photorespiration products, threonine is derived from the tricarboxylic acid (TCA) cycle, and valine from glycolytic pyruvate^44^. The dominance of these amino acids in the exponential growth phase thus indicates carbon and nitrogen acquisition, and can be used as a marker for actively growing *Phaeocystis* cells. The composition in free amino acids differed from other marine microalgae^23,24^, indicating taxon specificity in nitrogen assimilation and amino acid metabolism. Pyrrole-2-carboxylic acid, which is involved in pyrrole biosynthesis^45^, may be linked to amino acid metabolism. Even though this carboxylic acid is repeatedly found in microalgae^23,42^, its role in algal metabolism is unknown. Threonic acid, an oxidation product of ascorbic acid^46^, was also present at higher levels during exponential growth. As ascorbic acid is a well-known antioxidant in chloroplasts that scavenges reactive oxygen species, threonic acid may indicate elevated oxidative stress during high photosynthetic activity.

Characteristic for the exponential and even more the early stationary growth of *P. pouchetii* were mannitol and a quinic acid derivative. Mannitol is not produced by diatoms^47^, and represents a metabolic marker with certain taxon-specificity within phytoplankton blooms. Mannitol is the main short-term carbon storage molecule in *E. huxleyi*^48^, and may thus also play a role in carbon assimilation of *P. pouchetii*. It would indicate ongoing photosynthesis during growth limitation. The quinic acid derivative showed high mass spectral similarity to a quinic acid standard, however, retention indices were not alike (Supplementary Fig. S2). As quinic acid is related to the shikimate pathway fuelling the biosynthesis of aromatic amino acids and secondary metabolites, further characterization is recommended.

In the early stationary growth, mono- and disaccharides, sugar alcohols (pentitols), glycerol, and *scyllo*-inositol increased, reflecting an active carbohydrate metabolism. Pentitols may thereby indicate active interconversion of monosaccharide building blocks^49^, whereas glycerol can be linked to lipid metabolism^50^. The abundance of *scyllo*-inositol is interesting, as most commonly, *myo*-inositol is reported for algae, which was only present in small amounts and not regulated (data not shown). All eight inositol isomers are metabolized from *myo*-inositol and involved in e.g. phosphor storage^51^. The rare stereoisomer *scyllo*-inositol was reported for the haptophyte *Pavlova*, however, with unknown function^52^. Induced lipid metabolism during early and late stationary growth is further reflected by elevated levels of glyceraldehyde and methylated C18-polyunsaturated fatty acids (PUFAs). Glyceraldehyde can be derived from hexoses fuelling the TCA cycle and, via interconversion to glycerol, also the lipid metabolism. C18-PUFAs are synthesized from C18:0 via C18:1ω9^53^. *Phaeocystis* is known to contain high amounts of C18:0 and C18-PUFAs (especially C18:1ω9 and C18:5ω3) in the polar lipid fraction^54^, however, the occurrence and role of free fatty acids is poorly understood. The methylation of free fatty acid may be a storage artefact^55^. The observed increase in metabolites associated with lipid metabolism with the onset of stationary growth is in accordance with earlier studies on light, nutrient, and osmotic stress^56^.

In the late stationary growth, mono- and disaccharides, free fatty acids, sterols, tocopherols, and terpenes became abundant. These metabolites provide evidence for an active metabolism of carbohydrates, lipids and derived structures. Within the life cycle of *Phaeocystis*, monosaccharides play a role as precursors and intermediates in regular cell metabolism, whereas disaccharides are involved in the synthesis of polysaccharides for carbon storage, cell wall and mucus formation^6^. The observed intracellular accumulation of carbohydrates may reflect both the uncoupling of Calvin and TCA cycle during stationary phase, as well as an active metabolism of glucan, the main storage polysaccharide. Both, the dominant sterol of *P. pouchetii* 24-methylcholesta-5,22-dien-3β-ol^57^, and the membrane antioxidant α-tocopherol were most abundant in the late stationary phase. These metabolites are important for membrane stability towards declining growth^23,24^. The polyamine putrescine, which is a metabolic marker for the declining growth in diatoms and dinoflagellates^58,59^, was neither detected in *P. pouchetii* nor in *E. huxleyi* or other haptophytes^23,60^. This demonstrates that different strategies in metabolic stress response occur within algal lineages during growth decline.

Thus, the growth physiology of *P. pouchetii* is reflected in the levels of endometabolites, such as amino acids, fatty acids, and carbohydrates. Besides metabolite responses that are more general, such as for α-tocopherol, also responses with some taxon-specificity are observed, such as for mannitol.

### Physiological marker metabolites in natural plankton assemblages

Metabolite profiles of phytoplankton blooms reflect not only algal taxonomy, but also abiotic environmental factors and biotic interactions. Traditional chemotaxonomy approaches have recently been questioned, since the metabolic variability induced in response to environmental stressors might be more substantial than differences between species^22,61^. This study documents substantial physiological plasticity both in laboratory and in field experiments. Previous work linked fatty acids to trophic relationships and senescence^62,63^. Very few studies provide a direct link of metabolic variability in the field with algal performance, as demonstrated for the resistance to viral infection in *E. huxleyi*^64^. Our data allows surveying metabolic markers of growth phase-dependent physiology in the field using an unbiased metabolomics approach, and paves the way for a better understanding of phenotypic variability in plankton.

At all cruise stations, *Phaeocystis* was found and, in accordance, many of the endometabolites identified in laboratory cultures were detected in all investigated natural phytoplankton communities with variable concentrations. A diverse metabolic landscape shaped by the abundance of various plankton taxa in different growth phases as modulated by both biotic and abiotic factors was recognized (Fig. 5). Light attenuates with water depth, which has a strong impact on the photosynthetic activity of all algal cells as reflected by a hexose that decreased with greater depth (met. 281; Fig. 5b). The availability of nutrients such as NO_3_^−^ and PO_4_^3−^ strongly influences algal physiology and metabolism. Accordingly, distinct endometabolite profiles were observed in the nutrient-depleted inner Porsangerfjord (Station 4) with elevated levels of several mono- and disaccharides (Fig. 5c), as well as lipids and derived metabolites. This distinct pattern is likely a result of nutrient limitation and the resulting stress metabolism, as observed during the late growth phase in laboratory cultures. In addition, decline of a preceding diatom bloom as indicated by high bacterial production (0.31 µg C L^−1^ h^−1^) and abundance of heterotrophic flagellates (e.g. *Gyrodinium lachryma*) may have contributed to these metabolic profiles.

Several of the *P. pouchetii* endometabolites were most abundant at the stations dominated in biomass by *Phaeocystis* (Fig. 5e). Their differential distribution within these *Phaeocystis* blooms reflects different bloom physiologies. This is demonstrated e.g. by mannitol, which is not produced by diatoms^47^ and had an interesting distribution: it was lower at stations with high *Phaeocystis* biomass (> 50%, Station 5a/b) and higher at stations with low *Phaeocystis* biomass. In accordance with elevated mannitol levels during exponential and early stationary growth in the *P. pouchetii* cultures, this may indicate an early bloom phase with high carbon acquisition at Station 3. Stations 4–5 would correspond to later bloom phases of *P. pouchetii*. The high mannitol level at Station 6 at largest depth may be attributed to an elevated metabolic activity due to morphological changes: colony abundance increased with depth, cell sizes ranged from 2–6 µm with a high proportion of 6 µm cells (ca. 50%) and a relative increase of smaller cells with depth (Fig. 3). This indicates a late bloom stage at which aggregation and sinking of senescent *Phaeocystis* colonies induces the release of flagellated single cells^2^. Station 3 was also characterized by several free fatty acids reflecting active lipid metabolism, whereas Stations 5–6 showed high levels of the sterol 24-methylcholesta-5,22-dien-3β-ol. This sterol is a marker for late stationary growth in *Phaeocystis* laboratory cultures, and was relative to *Phaeocystis* biomass highest at the senescent Station 6 revealing again the presence of different bloom physiologies. A few metabolites were correlated either with the biomass of diatoms (e.g. EPA; Fig. 5d) or *Phaeocystis* (e.g. C18:1; Fig. 5e), and therefore rather reflect bloom taxonomy than physiology.

Laboratory-derived physiological marker metabolites thus supported the notion of different bloom stages as suggested by microscopy and FlowCam analysis. Their applicability seems to be robust despite the fact that in natural environments these laboratory-derived metabolites underlie additional constraints, such as complex microbial interactions or simply the effect of field sampling protocols with longer sample handling times.

## Conclusion

Taken together, the diverse metabolic landscape of natural plankton blooms was revealed and linked to the growth physiology of *P. pouchetii* using endometabolite markers of laboratory cultures. The physiological response of phytoplankton cells to irradiance, nutrient availability, and growth phase is reflected in their (meta)metabolite profiles both in laboratory cultures and field communities. Characterizing the physiological state of *Phaeocystis* blooms in addition to classical methods, such as microscopy or the targeted analysis of selected metabolites such as dimethylsulfoniopropionate, can give direct information on food quality, and may help to explain variability in zooplankton grazing. As cell physiology directly affects the metabolite composition of the algal phycosphere, it may also influence other species interactions including pathogens such as bacteria, viruses and fungi. Following cell lysis, the endometabolome is released to the dissolved organic matter pool, which may shape the marine microbial loop as well as the heterotrophic plankton community.

## Material and methods

### Solvents

Methanol, water, and pyridine (stored over 4 Å molecular sieve under argon) were purchased from Sigma-Aldrich. Chloroform (stabilized with ethanol) and tetrahydrofuran (for LC–MS, without stabilizer) as purchased from VWR were stored under argon. Ethanol in LC grade and hexane in GC grade were purchased from Merck. All other solvents had HPLC grade.

### Algal cultivation

*Phaeocystis pouchetii* AJ01 (isolated 1994 from Raunefjord, Norway) was obtained from Aud Larsen (Bergen University, Norway). The strain consists solely of diploid, flagellated cells, and showed no colony formation in culture since isolation^35^. Cultures were grown in sterile-filtered, autoclaved f/2 medium (-Si) based on natural seawater at 6.7 ± 0.9 °C, under a 14:10 h light:dark cycle, at 15–50 µmol photons s^−1^ m^−2^ provided by fluorescent tubes (Osram L15W/840 Lumilux Cool White). Cultures were shaken once daily. To adapt cells to experimental conditions, the stock culture was transferred in three subsequent passages within the exponential growth phase into fresh medium (10% ν:ν). In 2 L glass bottles, 1.62 L medium (n = 4) was inoculated with 180 mL adapted culture to reach an average initial cell abundance of 4.6 ± 3.3 × 10^3^ cells mL^−1^. An additional bottle with 1.8 L medium was used as medium control.

### Sampling of metadata

Every 2–3 days, 13 mL of culture was sampled to determine chlorophyll *a* (Chl *a*) fluorescence, photosystem II (PSII) efficiency, algal abundance, bacterial abundance and inorganic nutrient concentrations as previously described^23^. Briefly, Chl *a* fluorescence and PSII efficiency were determined with a plate reader in black well plates. F0 (initial fluorescence) was measured after incubation at 5 °C in the dark, and 30 s double orbital shaking. To determine algal abundance, samples were fixed with acidic Lugol's solution (1% final concentration), and stored at dark until investigation by light microscopy. At least 400 cells or at most 16 mm^2^ were counted in duplicates in a Fuchs-Rosenthal counting chamber at × 400 magnification. Growth rates were calculated by iterative fitting of the logistic growth model (${N}_{t}= \frac{K{N}_{0}{e}^{\mu t}}{K +{N}_{0}{(e}^{\mu t}-1)}$; *N* = population size, *t* = growth time, *K* = max. population size) to the observed abundances by minimizing their deviance using the SOLVER function in Excel. To determine bacterial abundance, aliquots of 1 mL were fixed with glutaraldehyde (0.5% final concentration), frozen in liquid nitrogen, and stored at − 80 °C until flow cytometry analysis. Thawed samples were diluted 1:50 with TE buffer (10 mM Tris–HCl, 1 mM EDTA, pH 8.0) and stained with SybrGold (Invitrogen, USA). Then, 100 µL stained sample was mixed with 300 µL TE buffer and 100 µL Fluoresbrite Plain YG 1.0 µm Microspheres (Polysciences, Germany), and immediately measured at 525 nm using a Cytomics FC 500 flow cytometer (Beckman Coulter, Germany) with a 20 mW 488 nm air-cooled argon-ion laser. Reference beads were calibrated with CountBright absolute counting beads (7 µm, Life technologies, USA) at 575 nm for 1 min with 10 µL min^−1^. To determine inorganic nutrient concentrations, aliquots of 10 mL were filtered (0.22 µm), fixed with chloroform (0.05% final concentration), and stored at − 20 °C until further analysis^24^. Concentrations of phosphate (PO_4_^3−^) and nitrite (NO_2_^−^) were determined colorimetrically^65,66^, whereas nitrate (NO_3_^−^) was analyzed using ion chromatography^67^.

### Sampling, extraction and derivatization of endometabolites

For endometabolite analysis, aliquots of 0.5 L were collected 2 h after onset of light from all cultures and the medium control following 12 days ('exp'), 26 days ('e.stat') and 47 days ('l.stat') of culturing. Cells were filtered through GF/C filters (47 mm diameter, Whatman) at 65 kPa under pressure with filtration times of ca. 20 min. Wet filters were immediately transferred to 25 mL glass beakers. A single filter was required to pass the entire culture volume from exponential cultures ('exp'), whereas two or three filters were required to pass the culture volume from early ('e.stat') and late ('l.stat') stationary cultures, respectively. After filtration, cells were immediately re-suspended in 1 mL extraction mix (methanol:ethanol:chloroform, 1:3:1, *v*: *v*: *v*) and, if multiple filters were used, combined in 4 mL glass vials. The time between filtration start and metabolite quenching in extraction mix was on average 20 min. As internal standard (IS), 5 µL ('l.stat': 10 µL) aqueous ribitol (4 mM; > 99%, Sigma-Aldrich) was added. Samples were kept at − 20 °C until analysis 1 month after the end of the experiment. For endometabolite profiling, samples (0.5 mL per filter) were transferred into 1.5 mL centrifuge tubes (Eppendorf, Germany), sonicated for 10 min, centrifuged for 15 min (30.000 g, 4 °C), and supernatants were transferred to 1.5 mL glass vials. Samples were evaporated to dryness under vacuum before subsequent derivatization with methoxyamine and MSTFA^24^. In case the IS peak was missing, samples were re-derivatized with 10 µL MSTFA and incubated for 30 min at 60 °C followed by 30 min at 40 °C.

### Gas chromatography-mass spectrometry (GC–MS) analysis

Samples were run on a Trace GC Ultra coupled to a ISQ LT and AS3000 II auto sampler (ThermoScientific) that was equipped with a DB-5 ms column (Agilent J&W, 30 m, 0.25 mm internal diameter, 0.25 µm film thickness, 10 m Duraguard pre-column) as previously described^24^. A new, deactivated glass liner (ThermoScientific, 5 × 8 × 105 mm inner × outer diameter × length) with glass wool was used for every batch of 21 samples. Samples were injected using split 10. The electron impact source was set to 70 eV at 280 °C. Resolution was 866 at *m*/*z* 502.20 (FWHM = 0.53).

### Data processing and statistical analysis

To allow background-noise correction using the MassLynx 4.1 CODA tool (Waters, MCQ = 0.8, Smoothing window = 5), all chromatograms were first converted to NetCDF with the Xcalibur File Converter (ThermoScientific) and then to RAW with the MassLynx 4.1 DataBridge (Waters). Signals were de-convoluted with AMDIS 2.71, and peaks were integrated with MET-IDEA 2.08 (instrument type: 'quadrupole)^24^, using the chromatogram with the highest number of components as model ion file. The resulting peak area table was imported in Microsoft Excel. All peaks were removed that corresponded to the internal standard, retention index (RI)-mixture, and contaminants (signals that were negative in all replicates of one day after subtraction of the corresponding solvent blank). Finally, data was peak sum normalized, and a canonical analysis of principal coordinates (CAP) was performed^24^. Variables (loadings) were screened for significant correlation with the first two CAP axes using the Pearson correlation test (k = 1, m = 2, α = 0.01). Score and loading plots were visualized in SigmaPlot version 11.0 (https://systatsoftware.com/products/sigmaplot/sigmaplot-how-to-cite-sigmaplot/ Systat Software, San Jose, CA) and heat maps in MetaboAnalyst 3.0^68^.

### Metabolite identification

Metabolite features (abbreviated as 'met.' and listed in Supplementary Table S6) were assigned to one of four identification levels^69^: metabolites, where both retention index and mass spectrum matched a measured chemical reference standard were classified as identified (level 1). Only these metabolites are mentioned and discussed by their name. Others were ranked according to their spectral similarity to a database compound, and annotated and discussed as putative compound (level 2) or compound class (level 3), or as unidentified (level 4). Chemical reference standards were derivatized as described above. Linear retention indices were calculated, and a match between detected metabolite and reference standard was accepted with *Δ*RI ≤ 26^70,71^. Mass spectra as extracted by AMDIS were manually compared to the following spectral databases using MS Search 2.0 g (Nist): NIST 11 library version (mainlib, replib, nist_ri), Golm Metabolome Database libraries T_MSRI_ID (https://csbdb.de/csbdb/dload/dl_msri.html; 2004) and GMD_20111121_VAR5_ALK_MSP (https://gmd.mpimp-golm.mpg.de/download/; 2011), and an in-house database (175 compounds from several metabolite classes including algal extracts of *Skeletonema marinoi*). Mass spectral annotations were regarded as match with a reverse match factor (R.Match) > 900, or tagged with '?' if the factor was 800–900 and '??' if it was 700–800. Metabolite data were deposited at the EMBL-EBI MetaboLights database (MTBLS758).

### Sampling of natural plankton communities

Phytoplankton communities were sampled in the Barents Sea and along the North Norwegian coast during the PHAEONIGMA cruise from 02–07 May 2013 on board of R/V *Håkon Mosby* (Table 1).

**Table 1:** Overview of sample location, date (dd/mm/yyyy) and water depth of all seven PHAEONIGMA stations within the Barents Sea and adjacent North Norwegian fjords.

#	Station description	Latitude	Longitude	Date	Sample depths
1	Arctic water	75° 48.15 N	20° 03.63 E	02/05/2013	5 m, 16 m, 31 m
2	Polar front	74° 42.51 N	19° 59.49 E	02/05/2013	6 m, 15 m, 30 m
3	Atlantic water	72° 45.50 N	20° 02.78 E	03/05/2013	6 m, 32 m, 58 m
4	Inner Porsangerfjord	70° 20.95 N	25° 15.57 E	04/05/2013	5 m, 20 m, 40 m
5a	Outer Porsangerfjord	70° 51.19 N	26° 05.36 E	04/05/2013	5 m, 20 m, 41 m
5b	Outer Porsangerfjord	70° 51.06 N	26° 04.70 E	06/05/2013	5 m, 20 m, 40 m
6	Ullsfjord	69° 55.79 N	19° 53.53 E	07/05/2013	5 m, 33 m, 50 m

At each station, vertical profiles of temperature, salinity, depth, oxygen, Chl *a* fluorescence, transmission and irradiance were measured with a CTD (Sea-Bird SBE 9) mounted to a rosette sampler with twelve 10 L Niskin bottles.

Samples for the analysis of dissolved inorganic nutrients and phytoplankton community composition were collected as previously described^72^. Briefly, nitrate (measured indirectly as nitrite following reduction), phosphate and silicate concentrations were determined colorimetrically using a continuous flow analyzer (Skalar San + Autoanalyzer) according to ISO17025^65^. For epifluorescence microscopy, a subsample of 50 mL was stained for 3 min with 20 µL primulin (250 µg mL^−1^ working solution with 0.1 M TRIS HCl at pH 4), fixed with glutaraldehyde (3.6% final concentration) and 70% glycerol (10*v*% final concentration), filtered onto black filters (Nucleopore, 0.4 µm), mounted on glass slides, and stored at − 20 °C until phytoplankton cell enumeration and carbon estimation^73^. In total, 234 taxa were identified and classified as either '*P. pouchetii*', 'diatom', 'flagellate', 'ciliate' or 'unknown cell'. In these counts, *Phaeocystis* was recorded as single cells in different size classes. Colonial cell abundance of *Phaeocystis* was determined for subsamples of 20 mL with a FlowCam IV (Fluid Imaging technologies, ME, USA) equipped with a × 4 objective and a FC300 flow cell (Fluid Imaging technologies)^74^. Colonial cell number was calculated from individual images of colonies by regression analysis^19^. To estimate the age of each *Phaeocystis* bloom, FlowCam images taken at the Chl *a* maximum were evaluated for size and structure (Supplementary Fig. S4): whereas intact colonies dominate early bloom stages, colonies in aging blooms develop typifying features such as the infestation by diatoms^73^ and appearance of flagellated cells during senescence^2^.

Water samples for metabolite analysis were collected above, within, and below the Chl *a* maximum (Table 1), and transferred to cleaned 10 L PE containers. Endometabolites were sampled in triplicates by filtering in parallel 2–6 L seawater at 60 kPa under pressure per single GF/C filter (47 mm diameter). Larger zooplankton was removed with a pipette. Cells were re-suspended in 1.2 mL extraction mix as described above. Filtration and extraction were performed in a 10 °C cold room. Average filtration time was 8–10 h and average time between sampling and quenching was ~ 12 h. Samples were stored at − 20 °C until transportation on ice to Jena, Germany, and at − 80 °C until GC–MS analysis 1 year later.

### GC–MS analysis of natural plankton samples

Samples were thawed, diluted with extraction mix to 1.7 mL, and 5 µL IS were added. Aliquots of 0.7 mL per sample were processed as described above. Derivatization was achieved in a reduced volume of 25 µL methoxymation solution and 25 µL silylation solution. Samples with low IS signal were re-derivatized with MSTFA as described above. GC–MS measurements were conducted using a 6890 N GC equipped with a 7683B auto sampler (Agilent) coupled to a Micromass GCT Premier (Waters) mass spectrometer. The GC was operated with glass liners (Agilent, 4 × 6.3 × 78.5 mm inner × outer diameter × length), split 1, and 250 °C injector temperature. The MS was used with 300 °C source temperature and dynamic range extension mode. Resolution was > 6.000 at *m*/*z* 501.97.

### Comparative data analysis of laboratory- and field-derived endometabolomes

To qualitatively compare the endometabolites of *P. pouchetii* strain AJ01 with those of the natural communities, all GC–MS raw files were converted to NetCDF and further processed with MetaboliteDetector 2.0^75^. Using 'RI Calibration Wizard', the peaks of the added IS and RI compounds were manually assigned to calibrate the system and calculate RI values for all deconvoluted features (for parameters see Supplementary Table S1, S2). This was done individually for the laboratory (including medium blanks) and the field data (including derivatization blanks). Using 'Batch quantification', the features of both data sets were quantified within all chromatograms (for parameters see Supplementary Table S3). The resulting file was exported to Microsoft Excel, features with < 3 matches within biological replicates, an average signal-to-noise ratio < 10, multiple extraction, or retention times outside the RI range were removed, and all remaining features manually verified based on RI and mass spectrum. A presence-absence data table for laboratory, field and control samples was generated and plotted as Venn diagram. Features that were shared between laboratory and field data, but which were absent in control samples, were visualized as semi-quantitative box plots using MetaboAnalyst 3.0, and as cruise transect section plots using Ocean Data View 4.7.10^76^ with DIVA gridding and automatic scale lengths. Spearman rank correlation coefficients, Monte Carlo-estimated p-values, and 95%-CI limits were calculated for selected metabolites and environmental parameters (Supplementary Table S4). In addition, a partial least squares (PLS) regression analysis was conducted using the R ‘pls’ package^77^ with the function ‘jackknife’ to test the correlation between these metabolites and environmental parameters. The influence of taxonomy, irradiance and nutrient availability was evaluated for all station samples; however, due to missing nutrient concentrations at Station 2 either this station or the three nutrient parameters were omitted. The influence of *Phaeocystis* physiology was evaluated for the five stations with *Phaeocystis* blooms omitting the taxonomic parameter ‘*Phaeocystis* presence’. For the Jackknife approximate *t* tests, regression models with at least one component were used.

## Supplementary information

Supplementary Information 1.

Supplementary Information 2.

## Data Availability

The laboratory dataset is available in the EMBL-EBI MetaboLights repository (MTBLS758). The environmental dataset is available from the corresponding author on reasonable request.
