# Supplementary material for: Metabolomics-derived marker metabolites to characterize Phaeocystis pouchetii physiology in natural plankton communities
Source: Sci Rep. 2020 Nov 24;10:20444. doi: 10.1038/s41598-020-77169-w (PMC7686483; doi:10.1038/s41598-020-77169-w)
Supplement: Supplementary file 1 — Supplementary Information 1. [file 41598_2020_77169_MOESM1_ESM.docx]

**Supplementary Information**

**Metabolomics-derived marker metabolites to characterize *Phaeocystis pouchetii* physiology in natural plankton communities**

Constanze Kuhlisch^1,a^, Julia Althammer^1,b^, Andrey F. Sazhin^2^, Hans H. Jakobsen^3^, Jens C. Nejstgaard^4^, Georg Pohnert^1*^

^1^ Institute for Inorganic and Analytical Chemistry, Friedrich Schiller University Jena, Lessingstraße 8, 07743 Jena, Germany

^2^ Shirshov Institute of Oceanology, Russian Academy of Sciences, Nakhimovsky Prospect 36, Moscow, Russia

^3^ Department of Bioscience, Aarhus University, Frederiksborgvej 399, 4000 Roskilde, Denmark

^4^ Leibniz-Institute of Freshwater Ecology and Inland Fisheries, Dep. 3, Alte Fischerhütte 2, 16775 Stechlin, Germany

^a^ Present address: Department of Plant and Environmental Sciences, Weizmann Institute of Science, 234 Herzl Street, 7610001 Rehovot, Israel

^b^ Present address: JenaBios GmbH, Löbstedter Straße 80, 07749 Jena, Germany

*corresponding author, e-mail: georg.pohnert@uni-jena.de

Supplementary Table S1 MetaboliteDetector 2.0: 'RI Calibration Wizard' advanced parameters.

| Calibration compound | Ribitol (5TMS) |
| --- | --- |
| Required score | 0.7 |

Supplementary Table S2 MetaboliteDetector 2.0: Deconvolution settings.

| Baseline adjustment | yes |
| --- | --- |
| Peak threshold | 10 |
| Minimal peak height | 10 |
| Bins/scan | 10 |
| Deconvolution width | 1 |

Supplementary Table S3 MetaboliteDetector 2.0: 'Batch quantification' parameters.

| Non-targeted analysis |  |
| --- | --- |
| Compound matching: |  |
| ΔRI | 15 |
| Pure/Impure | 0.7 |
| scoring method | RI + Spec |
| req. score | 0.5 |
| Identification: |  |
| ΔRI | 15 |
| Pure/Impure | 0.7 |
| Scoring Method | RI + Spec |
| Ref. library |  |
| Compound filter: |  |
| Compound reproducibility | 0.3 |
| Max. peak disc. index | 100 |
| Req. S/N | 0 |
| Min # ions | 10 |

Supplementary Table S4 Spearman rank correlation coefficients for selected *Phaeocystis pouchetii* endometabolites and environmental parameters for PHAEONIGMA cruise samples. Monte Carlo-estimated p-values and 95%-CI limits are given.

|  |  | Spearman rank correlation | | |
| --- | --- | --- | --- | --- |
| Environmental parameter | Endometabolite | Coefficient | p-value | 95%-CI |
| *Phaeocystis* biomass | *Scyllo*-Inositol | 0.644 | 0.002 | (0.396, 0.893) |
|  | Mannitol | 0.552 | 0.010 | (0.223, 0.881) |
| Diatom biomass | C20:5n-3 | 0.544 | 0.011 | (0.220, 0.869) |
| Irradiance | Hexose (met. 281) | 0.552 | 0.010 | (0.251, 0.853) |
| Nitrate concentration | Maltose | -0.512 | 0.030 | (-0.893, -0.131) |
|  | Ribose | -0.459 | 0.056 | (-0.943, 0.026) |

**Supplementary Table S5** Partial least squares regression analysis for selected *Phaeocystis pouchetii* endometabolites and environmental parameters for PHAEONIGMA cruise samples. n_var_ – number of tested environmental parameters. % variance – cumulative explained variance (in %) for components 1-3. n_comp_ – number of components needed based on adjCV of RMSEP. Only the environmental variables with a significant correlation following a Jackknife approximate *t* test are presented: est. coeff. – estimated regression coefficient, S.E. – standard error of regression coefficient, df – degrees of freedom.

|  | **Metabolite^1^** | **n_var_** | **% variance** | | **n_comp_** | **Variable^2^** | **Est. coeff.** | **S. E.** | **df** | **t value** | **p value** |  |
| --- | --- | --- | --- | --- | --- | --- | --- | --- | --- | --- | --- | --- |
| Irradiance | Hexose (met. 281) | 13 | 1^st^ | 22.9 | 1 | Phaeocystis-%BM | -9.7E-04 | 3.0E-04 | 20 | -3.17 | 0.005 | ** |
|  |  |  | 2^nd^ | 35.1 |  | Diatoms-%BM | 1.2E-03 | 4.9E-04 | 20 | 2.52 | 0.020 | * |
|  |  |  | 3^rd^ | 42.9 |  | Unknown-BM | 1.5E-03 | 7.2E-04 | 20 | 2.12 | 0.047 | * |
|  |  |  |  |  |  |  |  |  |  |  |  |  |
|  |  |  |  |  |  |  |  |  |  |  |  |  |
| Nutrient conc. | Maltose | 16 | 1^st^ | 43.2 | 0 | Nitrate | -7.2E-05 | 3.8E-05 | 17 | -1.87 | 0.078 | . |
|  |  |  | 2^nd^ | 61.4 |  |  |  |  |  |  |  |  |
|  |  |  | 3^rd^ | 72.5 |  |  |  |  |  |  |  |  |
|  | Ribose | 16 | 1^st^ | 23.8 | 0 | Silicate | -1.8E-04 | 1.0E-04 | 17 | -1.80 | 0.090 | . |
|  |  |  | 2^nd^ | 45.5 |  | Nitrate | -1.5E-04 | 8.2E-05 | 17 | -1.79 | 0.091 | . |
|  |  |  | 3^rd^ | 62.0 |  |  |  |  |  |  |  |  |
| Taxonomy (Diatoms vs. *Phaeocystis*) | C20:5n-3 | 13 | 1^st^ | 34.7 | 1 | Diatoms-%BM | 1.9E-04 | 6.7E-05 | 20 | 2.80 | 0.011 | * |
|  |  |  | 2^nd^ | 46.9 |  | Phaeocystis-%BM | -1.7E-04 | 6.0E-05 | 20 | -2.79 | 0.011 | * |
|  |  |  | 3^rd^ | 50.2 |  | Salinity | 1.9E-04 | 7.7E-05 | 20 | 2.45 | 0.024 | * |
|  |  |  |  |  |  | Phaeo-presence | -1.4E-04 | 7.2E-05 | 20 | -2.03 | 0.056 | . |
|  | met. 343 | 13 | 1^st^ | 51.1 | 1 | BiomassPp% | -2.9E-03 | 6.9E-04 | 20 | -4.18 | 0.000 | *** |
|  |  |  | 2^nd^ | 63.1 |  | Diatoms-%BM | 3.1E-03 | 7.4E-04 | 20 | 4.10 | 0.001 | *** |
|  |  |  | 3^rd^ | 70.7 |  | Phaeo-presence | -2.2E-03 | 5.4E-04 | 20 | -3.97 | 0.001 | *** |
|  |  |  |  |  |  | Diatoms-BM | 1.8E-03 | 4.8E-04 | 20 | 3.85 | 0.001 | ** |
|  |  |  |  |  |  | Unknown-BM | 1.6E-03 | 4.5E-04 | 20 | 3.66 | 0.002 | ** |
|  |  |  |  |  |  | Salinity | 2.8E-03 | 1.1E-03 | 20 | 2.49 | 0.021 | * |
|  |  |  |  |  |  | Flagellates-BM | 3.3E-03 | 1.6E-03 | 20 | 2.10 | 0.048 | * |
|  |  |  |  |  |  | Temperature | -1.7E-03 | 8.1E-04 | 20 | -2.07 | 0.051 | . |
|  |  |  |  |  |  | Depth | -1.6E-03 | 8.5E-04 | 20 | -1.90 | 0.072 | . |
|  | C18:1 | 13 | 1^st^ | 38.1 | 1 | Temperature | 7.9E-06 | 2.7E-06 | 20 | 2.99 | 0.007 | ** |
|  |  |  | 2^nd^ | 56.6 |  | Unknown-BM | -5.3E-06 | 1.8E-06 | 20 | -2.98 | 0.007 | ** |
|  |  |  | 3^rd^ | 64.2 |  | Phaeo-presence | 5.8E-06 | 2.2E-06 | 20 | 2.60 | 0.017 | * |
|  |  |  |  |  |  | Phaeocystis-BM | 1.1E-05 | 4.4E-06 | 20 | 2.58 | 0.018 | * |
|  |  |  |  |  |  | Phaeocystis-%BM | 6.8E-06 | 3.9E-06 | 20 | 1.74 | 0.097 | . |
| *Phaeocystis* physiology | C22:6n-3 | 15 | 1^st^ | 61.3 | 2 | Flagellates-BM | 5.2E-05 | 1.7E-05 | 14 | 3.11 | 0.008 | ** |
|  |  |  | 2^nd^ | 71.9 |  | Temperature | 6.1E-05 | 2.7E-05 | 14 | 2.30 | 0.037 | * |
|  |  |  | 3^rd^ | 80.8 |  | Light | 8.1E-05 | 3.7E-05 | 14 | 2.15 | 0.049 | * |
|  |  |  |  |  |  | Ciliates-BM | 7.8E-05 | 4.3E-05 | 14 | 1.80 | 0.093 | . |
|  | Mannitol | 15 | 1^st^ | 23.3 | 0 | Diatoms-BM | 9.4E-04 | 5.0E-04 | 14 | 1.89 | 0.080 | . |
|  |  |  | 2^nd^ | 51.1 |  |  |  |  |  |  |  |  |
|  |  |  | 3^rd^ | 61.1 |  |  |  |  |  |  |  |  |
|  | *Scyllo*-inositol | 15 | 1^st^ | 58.1 | 2 | Ciliates-BM | 4.7E-03 | 2.3E-03 | 14 | 2.03 | 0.062 | . |
|  |  |  | 2^nd^ | 75.0 |  |  |  |  |  |  |  |  |
|  |  |  | 3^rd^ | 81.4 |  |  |  |  |  |  |  |  |
|  | 24-Methylcholesta-  5,22-dien-3β-ol | 15 | 1^st^ | 46.4 | 3 | Diatoms-%BM | -4.0E-04 | 1.9E-04 | 14 | -2.08 | 0.056 | . |
|  |  |  | 2^nd^ | 63.9 |  |  |  |  |  |  |  |  |
|  |  |  | 3^rd^ | 73.8 |  |  |  |  |  |  |  |  |
|  | C18:0 | 15 | 1^st^ | 48.4 | 1 | Silicate | 7.9E-05 | 2.0E-05 | 14 | 4.03 | 0.001 | ** |
|  |  |  | 2^nd^ | 65.9 |  | Salinity | 5.8E-05 | 2.5E-05 | 14 | 2.34 | 0.035 | * |
|  |  |  | 3^rd^ | 79.5 |  | Temperature | 7.4E-05 | 3.2E-05 | 14 | 2.30 | 0.037 | * |
|  | C18:4n-3 | 15 | 1^st^ | 49.2 | 2 | - | - | - | - | - | - |  |
|  |  |  | 2^nd^ | 63.3 |  |  |  |  |  |  |  |  |
|  |  |  | 3^rd^ | 77.5 |  |  |  |  |  |  |  |  |
|  | 1-C18:0-glycerol | 15 | 1^st^ | 31.3 | 0 | Nitrate | -3.2E-05 | 1.8E-05 | 14 | -1.80 | 0.093 | . |
|  |  |  | 2^nd^ | 55.9 |  |  |  |  |  |  |  |  |
|  |  |  | 3^rd^ | 66.1 |  |  |  |  |  |  |  |  |

*1 – Selected metabolites that occurred in both* Phaeocystis pouchetii *cultures and natural phytoplankton blooms (Figure 5). 2 – Putatively explanatory environmental variables (n = 16) that were tested for correlation included: depth, irradiance, temperature, salinity, chlorophyll a, absolute biomass of phytoplankton taxa (*Phaeocystis*, diatoms, flagellates, ciliates, unknown), relative biomass of diatoms and* Phaeocystis, *presence of* Phaeocystis, *nutrient concentrations (nitrate, phosphate, silicate).*

Supplementary Table S6 Intracellular metabolites that are highly correlated (x ≥0.8) with the exponential ('exp'), early stationary ('e.stat') or late stationary ('l.stat') growth phase of *Phaeocystis pouchetii* AJ01 (n = 4). # - MetIdea feature numbers as tabulated in Figure 2b. Ion - model ion used for peak integration. t_R_ - retention time (min). Vector - correlation coefficient. Factor - ratio of maximum to minimum growth phase median. Phase - correlated growth phase. R.Match - parameter of spectral database comparison (MS Search). (r) I_L_ - (reference) linear retention index. Level - identification level according to Sumner *et al.* (2007).

| **#** | **Ion** | **t_R_** | **Vector** | **A1** | **A2** | **A3** | **A4** | **B1** | **B2** | **B3** | **B4** | **C1** | **C2** | **C3** | **C4** | **Factor** | **Phase** | **Class** | **Substance name** | **R.Match** | **I_L_** | **r I_L_** | **Level** |
| --- | --- | --- | --- | --- | --- | --- | --- | --- | --- | --- | --- | --- | --- | --- | --- | --- | --- | --- | --- | --- | --- | --- | --- |
| 22 | 114.9 | 5.454 | 0.8232 | 0.0004 | 0.0002 | 0.0005 | 0.0004 | 0.0000 | 0.0000 | 0.0002 | 0.0000 | 0.0002 | 0.0000 | 0.0000 | 0.0003 | 10 | exp | Unknown | - | <700 | 1007 | - | 4 |
| 24 | 117 | 5.534 | 0.9144 | 0.0003 | 0.0002 | 0.0003 | 0.0002 | 0.0011 | 0.0010 | 0.0003 | 0.0008 | 0.0013 | 0.0009 | 0.0011 | 0.0005 | 3 | l.stat | Unknown | - | <700 | 1015 | - | 4 |
| 27 | 160 | 5.715 | 0.8538 | 0.0004 | 0.0002 | 0.0004 | 0.0003 | 0.0000 | 0.0000 | 0.0003 | 0.0000 | 0.0001 | 0.0000 | 0.0000 | 0.0000 | 8 | exp | Unknown | - | <700 | 1034 | - | 4 |
| 34 | 151 | 5.953 | 0.8759 | 0.0039 | 0.0044 | 0.0040 | 0.0037 | 0.0076 | 0.0048 | 0.0045 | 0.0114 | 0.0128 | 0.0150 | 0.0568 | 0.0148 | 6 | l.stat | Others | Phenol (1TMS) | 918 | 1059 | - | 2 |
| 36 | 242.3 | 6.034 | 0.8756 | 0.0000 | 0.0001 | 0.0000 | 0.0000 | 0.0000 | 0.0000 | 0.0000 | 0.0000 | 0.0000 | 0.0000 | 0.0000 | 0.0000 | 24 | exp | Unknown | - | <700 | 1067 | - | 4 |
| 38 | 173.1 | 6.122 | 0.9313 | 0.0009 | 0.0007 | 0.0008 | 0.0006 | 0.0010 | 0.0004 | 0.0010 | 0.0007 | 0.0015 | 0.0029 | 0.0057 | 0.0028 | 3 | l.stat | Fatty acid | Hexanoic acid (1TMS) | 719 | 1076 | 1075 | 3 |
| 42 | 145.9 | 6.315 | 0.9374 | 0.0001 | 0.0001 | 0.0001 | 0.0002 | 0.0000 | 0.0000 | 0.0001 | 0.0000 | 0.0000 | 0.0000 | 0.0000 | 0.0001 | 6 | exp | Amino acid | Valine (1TMS) | 798 | 1096 | 1093 | 1 |
| 47 | 116 | 6.408 | 0.9098 | 0.0034 | 0.0041 | 0.0044 | 0.0033 | 0.0001 | 0.0001 | 0.0026 | 0.0000 | 0.0001 | 0.0001 | 0.0000 | 0.0002 | 62 | exp | Amino acid | Alanine (2TMS) | 841 | 1106 | 1103 | 1 |
| 83 | 103.1 | 7.561 | 0.8763 | 0.0003 | 0.0003 | 0.0005 | 0.0003 | 0.0021 | 0.0012 | 0.0009 | 0.0014 | 0.0019 | 0.0008 | 0.0024 | 0.0004 | 5 | e./l. stat | Saccharide | Glyceraldehyde (Meox 2TMS) | 819 | 1226 | 1224 | 1 |
| 86 | 110 | 7.787 | 0.8198 | 0.0145 | 0.0102 | 0.0206 | 0.0115 | 0.0026 | 0.0050 | 0.0058 | 0.0031 | 0.0020 | 0.0007 | 0.0000 | 0.0009 | 17 | exp | Others | 1-Methyl-6-pyrimidinone | 749 | 1249 | - | 3 |
| 95 | 132 | 7.968 | 0.8287 | 0.0008 | 0.0008 | 0.0007 | 0.0005 | 0.0000 | 0.0000 | 0.0002 | 0.0000 | 0.0001 | 0.0000 | 0.0001 | 0.0000 | 50 | exp | Amino acid | Serine (2TMS) | 777 | 1268 | 1265 | 1 |
| 96 | 153.1 | 7.985 | 0.8102 | 0.0010 | 0.0004 | 0.0006 | 0.0005 | 0.0001 | 0.0000 | 0.0005 | 0.0001 | 0.0001 | 0.0000 | 0.0000 | 0.0001 | 11 | exp | Unknown | - | <700 | 1270 | - | 4 |
| 98 | 117 | 8.063 | 0.8306 | 0.0293 | 0.0157 | 0.0159 | 0.0195 | 0.0342 | 0.0230 | 0.0281 | 0.0258 | 0.0151 | 0.0141 | 0.0113 | 0.0139 | 2 | e.stat | Sugar alcohol | Glycerol (3TMS) | 915 | 1278 | 1277 | 1 |
| 105 | 117 | 8.314 | 0.8415 | 0.0014 | 0.0010 | 0.0009 | 0.0012 | 0.0003 | 0.0001 | 0.0004 | 0.0002 | 0.0001 | 0.0001 | 0.0001 | 0.0002 | 8 | exp | Amino acid | Threonine (2TMS) | 821 | 1304 | 1300 | 1 |
| 109 | 174 | 8.434 | 0.8116 | 0.0004 | 0.0012 | 0.0009 | 0.0003 | 0.0000 | 0.0000 | 0.0003 | 0.0000 | 0.0000 | 0.0001 | 0.0000 | 0.0001 | 38 | exp | Amino acid | Glycine (3TMS) | 819 | 1316 | 1313 | 1 |
| 118 | 166 | 8.855 | 0.8510 | 0.0001 | 0.0001 | 0.0001 | 0.0001 | 0.0000 | 0.0000 | 0.0000 | 0.0000 | 0.0001 | 0.0000 | 0.0000 | 0.0000 | 8 | exp | Others | Pyrrole-2-carboxylic acid (2TMS) | 818 | 1360 | 1357 | 1 |
| 121 | 162 | 8.927 | 0.8454 | 0.0004 | 0.0003 | 0.0005 | 0.0005 | 0.0001 | 0.0001 | 0.0004 | 0.0000 | 0.0001 | 0.0000 | 0.0000 | 0.0000 | 17 | exp | Unknown | - | <700 | 1368 | - | 4 |
| 122 | 188.1 | 8.939 | 0.8704 | 0.0027 | 0.0043 | 0.0053 | 0.0054 | 0.0032 | 0.0035 | 0.0048 | 0.0044 | 0.0028 | 0.0020 | 0.0000 | 0.0025 | 2 | exp | Amino acid | Alanine (3TMS) | 905 | 1369 | 1366 | 1 |
| 123 | 110 | 8.994 | 0.8037 | 0.0074 | 0.0031 | 0.0079 | 0.0045 | 0.0005 | 0.0004 | 0.0029 | 0.0002 | 0.0002 | 0.0001 | 0.0000 | 0.0000 | 108 | exp | Unknown | - | <700 | 1375 | - | 4 |
| 130 | 143 | 9.175 | 0.8111 | 0.0005 | 0.0005 | 0.0003 | 0.0004 | 0.0002 | 0.0001 | 0.0005 | 0.0001 | 0.0002 | 0.0004 | 0.0000 | 0.0003 | 2 | exp | Unknown | - | <700 | 1393 | - | 4 |
| 150 | 232.2 | 9.882 | 0.8658 | 0.0002 | 0.0001 | 0.0002 | 0.0002 | 0.0000 | 0.0000 | 0.0000 | 0.0000 | 0.0000 | 0.0000 | 0.0000 | 0.0000 | 36 | exp | Unknown | Pyruvic acid oxime (2TMS) or Aspartic acid (3TMS) | 747 | 1467 | - | 4 |
| 155 | 109.1 | 10.018 | 0.8887 | 0.0002 | 0.0001 | 0.0001 | 0.0001 | 0.0001 | 0.0001 | 0.0001 | 0.0001 | 0.0003 | 0.0003 | 0.0004 | 0.0002 | 4 | l.stat | Alcohols | Pentadecanal/Decanediol/ Dodecenol | 758 | 1481 | - | 3 |
| 159 | 228.1 | 10.101 | 0.8764 | 0.0003 | 0.0003 | 0.0002 | 0.0003 | 0.0001 | 0.0001 | 0.0002 | 0.0000 | 0.0000 | 0.0000 | 0.0000 | 0.0000 | 14 | exp | Unknown | - | <700 | 1490 | - | 4 |
| 165 | 241.9 | 10.191 | 0.8355 | 0.0001 | 0.0001 | 0.0002 | 0.0001 | 0.0000 | 0.0000 | 0.0001 | 0.0000 | 0.0000 | 0.0000 | 0.0000 | 0.0000 | 114 | exp | Unknown | - | <700 | 1499 | - | 4 |

The full list is provided in a separate excel document (tab 'Table S6').

Supplementary Table S7 PHAEONIGMA cruise metadata including station details, nutrient concentrations and phytoplankton measurements sampled along a transect in the Barents Sea (Station 1-3) and in North Norwegian fjords (Station 4-6) above, at and below the Chl *a* maximum. Station # - identifier used throughout the manuscript. Station log - identifier as logged during the cruise.

| **Station parameters** | | | | | | **Nutrient concentrations** | | | **Phytoplankton biomass** | | | | | ***P.pouchetii* cell size biomass** | | | | | ***P. pouchetii*** |
| --- | --- | --- | --- | --- | --- | --- | --- | --- | --- | --- | --- | --- | --- | --- | --- | --- | --- | --- | --- |
| **Station** | **Station** | **Description** | **Longitude** | **Latitude** | **Depth** | **NO3** | **PO4** | **Si** | ***P. pouchetii*** | **Diatoms** | **Flagellates** | **Ciliates** | **Unknown** | **2 µm** | **3 µm** | **4 µm** | **5 µm** | **6 µm** | **colonial cells** |
| **#** | **log** |  | **N** | **E** | **m** | **μmol/l** | **μmol/l** | **μmol/l** | **mg/m²** | **mg/m²** | **mg/m²** | **mg/m²** | **mg/m²** | **mg/m²** | **mg/m²** | **mg/m²** | **mg/m²** | **mg/m²** | **cells/mL** |
| 1 | 569 | Arctic Water | 75° 48.15' | 20° 03.63' | 5 | 4.229 | 0.339 | 1.616 | 50 | 910 | 146 | 4 | 81 | 0 | 3 | 4 | 43 | 0 | 15 |
| 1 | 569 | Arctic Water | 75° 48.15' | 20° 03.63' | 15 | 3.664 | 0.296 | 1.241 | 9 | 4177 | 258 | 14 | 40 | 0 | 1 | 2 | 6 | 0 | 16 |
| 1 | 569 | Arctic Water | 75° 48.15' | 20° 03.63' | 30 | 3.921 | 0.303 | 1.241 | 5 | 5528 | 186 | 3 | 15 | 0 | 1 | 3 | 1 | 0 | 18 |
| 2 | 571 | Polar Front | 74° 42.51' | 19° 59.49' | 5 | n.a. | n.a. | n.a. | 9 | 465 | 53 | 39 | 22 | 0 | 1 | 0 | 7 | 0 | 135 |
| 2 | 571 | Polar Front | 74° 42.51' | 19° 59.49' | 15 | n.a. | n.a. | n.a. | 6 | 596 | 32 | 4 | 15 | 0 | 1 | 1 | 4 | 0 | 169 |
| 2 | 571 | Polar Front | 74° 42.51' | 19° 59.49' | 30 | n.a. | n.a. | n.a. | 5 | 147 | 14 | 5 | 6 | 0 | 0 | 1 | 3 | 0 | 109 |
| 3 | 572 | Atlantic Water | 72° 45.50' | 20° 02.78' | 5 | 5.861 | 0.531 | 1.963 | 363 | 1397 | 106 | 71 | 15 | 0 | 0 | 0 | 362 | 0 | 7068 |
| 3 | 572 | Atlantic Water | 72° 45.50' | 20° 02.78' | 30 | 8.317 | 0.490 | 3.384 | 289 | 1121 | 260 | 45 | 8 | 0 | 1 | 0 | 288 | 0 | 2668 |
| 3 | 572 | Atlantic Water | 72° 45.50' | 20° 02.78' | 60 | 8.645 | 0.602 | 3.040 | 193 | 525 | 65 | 15 | 3 | 0 | 0 | 1 | 192 | 0 |  |
| 4 | 575 | Inner Porsangerfjord | 70° 20.95' | 25° 15.57' | 5 | 0.000 | 0.000 | 0.118 | 274 | 395 | 99 | 18 | 12 | 0 | 1 | 1 | 271 | 0 | 12516 |
| 4 | 575 | Inner Porsangerfjord | 70° 20.95' | 25° 15.57' | 20 | 0.000 | 0.051 | 0.223 | 300 | 557 | 92 | 12 | 37 | 0 | 1 | 1 | 298 | 0 | 18427 |
| 4 | 575 | Inner Porsangerfjord | 70° 20.95' | 25° 15.57' | 40 | 0.495 | 0.020 | 0.309 | 328 | 448 | 32 | 12 | 21 | 0 | 0 | 0 | 327 | 0 |  |
| 5a | 576 | Outer Porsangerfjord | 70° 51.19' | 26° 05.36' | 5 | 0.091 | 0.109 | 1.114 | 444 | 66 | 52 | 246 | 15 | 0 | 0 | 1 | 443 | 0 | 30620 |
| 5a | 576 | Outer Porsangerfjord | 70° 51.19' | 26° 05.36' | 20 | 3.431 | 0.225 | 1.321 | 376 | 53 | 38 | 166 | 4 | 0 | 0 | 0 | 375 | 0 | 15616 |
| 5a | 576 | Outer Porsangerfjord | 70° 51.19' | 26° 05.36' | 40 | 5.071 | 0.335 | 1.787 | 241 | 25 | 17 | 62 | 3 | 0 | 0 | 0 | 240 | 0 | 3016 |
| 5b | 584 | Outer Porsangerfjord | 70° 51.06' | 26° 04.70' | 5 | 0.674 | 0.062 | 1.557 | 629 | 131 | 34 | 34 | 7 | 0 | 1 | 1 | 627 | 0 | 32835 |
| 5b | 584 | Outer Porsangerfjord | 70° 51.06' | 26° 04.70' | 20 | 2.099 | 0.235 | 0.811 | 490 | 221 | 12 | 13 | 4 | 0 | 1 | 1 | 488 | 0 | 18845 |
| 5b | 584 | Outer Porsangerfjord | 70° 51.06' | 26° 04.70' | 40 | 3.709 | 0.275 | 0.933 | 345 | 87 | 17 | 11 | 3 | 0 | 1 | 1 | 343 | 0 | 5557 |
| 6 | 586 | Ullsfjord | 69° 55.79' | 19° 53.53' | 5 | 0.000 | 0.000 | 1.054 | 29 | 4 | 32 | 22 | 11 | 2 | 6 | 3 | 5 | 14 | 1286 |
| 6 | 586 | Ullsfjord | 69° 55.79' | 19° 53.53' | 38 | 3.197 | 0.247 | 1.036 | 37 | 7 | 27 | 13 | 15 | 4 | 9 | 3 | 4 | 17 | 3663 |
| 6 | 586 | Ullsfjord | 69° 55.79' | 19° 53.53' | 50 | 4.358 | 0.287 | 1.628 | 24 | 1 | 16 | 14 | 5 | 3 | 7 | 3 | 3 | 9 | 6358 |

The data is also provided in a separate excel document (tab 'Table S7').

Supplementary Table S8 Normalized abundance (peak area/peak sum) of intracellular metabolites of *Phaeocystis pouchetii* laboratory cultures (n = 4) in natural plankton communities sampled at station 1-6 during the PHAEONIGMA cruise (3 depths with n = 3). # - Metabolites are shown as tabulated in Figure 2b. Phase - correlated growth phase (exponential phase (exp), early stationary phase (e.stat), late stationary phase (l.stat), not regulated (-)). Cluster - respective cluster according to Supplementary Figure S5. Substance name - Metabolite name indicating MS Search match via "?" and modification of functional groups (TMS - trimethylsilyl, Meox - methoxyamine, Me - methyl). Level - identification level according to Sumner *et al.* (2007).

| Lab data | | Field data | | Metabolite identification | | | Station 1 | | | | | | | | Station 2 | | | | | | | |
| --- | --- | --- | --- | --- | --- | --- | --- | --- | --- | --- | --- | --- | --- | --- | --- | --- | --- | --- | --- | --- | --- | --- |
| # | Phase | # | Cluster | Class | Substance name | Level | 1a | 1b | 1c | 2a | 2b | 2c | 3a | 3b | 1a | 1b | 2a | 2b | 2c | 3a | 3b | 3c |
| 286 | e.stat | 240 | 1 | Fatty acid | C16:0 (Me) | 1 | 0.000711 | 0.000462 | 0.002043 | 0.000497 | 0.000395 | 0.001473 | 0.003162 | 0.000023 | 0.000090 | 0.001502 | 0.000297 | 0.003973 | 0.003402 | 0.007533 | 0.000617 | 0.000172 |
| M | e.stat | 281 | 1 | Fatty acid | C18:0 (Me) | 1 | 0.000121 | 0.000018 | 0.000120 | 0.000037 | 0.000036 | 0.000041 | 0.000415 | 0.000000 | 0.000007 | 0.000154 | 0.000046 | 0.000746 | 0.000654 | 0.001395 | 0.000071 | 0.000049 |
| 333 | - | 279 | 1 | Fatty acid | C18:1 (Me) | 1 | 0.000011 | 0.000004 | 0.000112 | 0.000011 | 0.000009 | 0.000000 | 0.000111 | 0.000000 | 0.000004 | 0.000049 | 0.000013 | 0.000131 | 0.000094 | 0.000192 | 0.000023 | 0.000009 |
| 327 | e./l.stat | 274 | 1 | Fatty acid | C18:4n-3 (Me) | 1 | 0.000504 | 0.000186 | 0.001930 | 0.000314 | 0.000343 | 0.001639 | 0.002167 | 0.000088 | 0.000018 | 0.001082 | 0.000163 | 0.001490 | 0.001584 | 0.002454 | 0.000393 | 0.000036 |
| 395 | e./l.stat | 339 | 1 | Fatty acid | C22:6n-3 (Me) | 1 | 0.000190 | 0.000030 | 0.000316 | 0.000148 | 0.000193 | 0.000840 | 0.001287 | 0.000051 | 0.000000 | 0.000452 | 0.000089 | 0.000579 | 0.000597 | 0.000953 | 0.000213 | 0.000000 |
| M | l.stat | 386 | 1 | FA derivative | 1-C18:0-glycerol (2TMS) | 1 | 0.000359 | 0.000000 | 0.001088 | 0.000381 | 0.000356 | 0.001845 | 0.001256 | 0.000000 | 0.000051 | 0.001425 | 0.000123 | 0.000727 | 0.000460 | 0.001288 | 0.000600 | 0.000000 |
| 473 | l.stat | 416 | 1 | Sterol | 24-Methylcholesta-5,22-dien-3beta-ol (1TMS) | 1 | 0.000263 | 0.000051 | 0.001248 | 0.000751 | 0.001013 | 0.003087 | 0.003836 | 0.000006 | 0.000191 | 0.003444 | 0.000199 | 0.001271 | 0.001519 | 0.002346 | 0.000810 | 0.000004 |
| 284 | exp/e.stat | 239 | 1 | Sugar alcohol | Mannitol (6TMS) | 1 | 0.000606 | 0.002192 | 0.000000 | 0.001289 | 0.001558 | 0.003310 | 0.002578 | 0.000000 | 0.000899 | 0.005292 | 0.000546 | 0.000000 | 0.003534 | 0.000000 | 0.001715 | 0.000000 |
| 311 | e.stat | 262 | 1 | Sugar alcohol | Scyllo-Inositol (6TMS) | 1 | 0.000288 | 0.001364 | 0.000000 | 0.001667 | 0.001769 | 0.004872 | 0.005703 | 0.000000 | 0.000952 | 0.005263 | 0.000347 | 0.002599 | 0.000000 | 0.007715 | 0.001710 | 0.000000 |
| M | - | 72 | 1 | Unknown | - | 4 | 0.000000 | 0.000000 | 0.000000 | 0.000005 | 0.000005 | 0.000000 | 0.000000 | 0.000000 | 0.000007 | 0.000046 | 0.000000 | 0.000000 | 0.000000 | 0.000000 | 0.000000 | 0.000000 |
| 172 | - | 147 | 1 | Unknown | - | 4 | 0.000628 | 0.001118 | 0.000901 | 0.000685 | 0.000717 | 0.001991 | 0.001860 | 0.000129 | 0.000302 | 0.003868 | 0.000164 | 0.000985 | 0.001475 | 0.001327 | 0.000882 | 0.000201 |
| M | l.stat | 338 | 1 | Unknown | - | 4 | 0.000080 | 0.000000 | 0.000111 | 0.000059 | 0.000056 | 0.000221 | 0.000194 | 0.000000 | 0.000025 | 0.000514 | 0.000024 | 0.000284 | 0.000401 | 0.000280 | 0.000115 | 0.000009 |
| 253 | - | 215 | 1 | Unknown | ? Skel_Media_C168/205 | 4 | 0.000910 | 0.001304 | 0.001893 | 0.001593 | 0.001608 | 0.003566 | 0.004956 | 0.000000 | 0.000581 | 0.008078 | 0.000476 | 0.000000 | 0.002332 | 0.002969 | 0.002369 | 0.000300 |
| M | l.stat | 97 | 1 | Unknown | ?? Skel_Media_C086 | 4 | 0.000762 | 0.001254 | 0.002352 | 0.001210 | 0.001257 | 0.001776 | 0.003494 | 0.000001 | 0.000002 | 0.005586 | 0.000402 | 0.001718 | 0.002768 | 0.004111 | 0.001403 | 0.000004 |
| 314 | - | 261 | 2 | Fatty acid | C16:1 (1TMS) | 1 | 0.000000 | 0.000000 | 0.000000 | 0.000434 | 0.000811 | 0.000000 | 0.002842 | 0.000000 | 0.001298 | 0.002348 | 0.000455 | 0.000000 | 0.000000 | 0.001613 | 0.002083 | 0.000202 |
| 332 | - | 278 | 2 | Fatty acid | ? C18:1/C18:3 (Me) | 2 | 0.000000 | 0.000000 | 0.000000 | 0.000082 | 0.000087 | 0.000000 | 0.000299 | 0.000000 | 0.000059 | 0.000112 | 0.000037 | 0.000000 | 0.000000 | 0.000058 | 0.000105 | 0.000000 |
| 383 | - | 330 | 2 | FA derivative | 1-C14:0-glycerol (2TMS) | 1 | 0.000318 | 0.000510 | 0.002677 | 0.001450 | 0.001080 | 0.003698 | 0.003832 | 0.000000 | 0.000200 | 0.001281 | 0.000182 | 0.000480 | 0.000185 | 0.001297 | 0.000725 | 0.000057 |
| 410 | e./l.stat | 358 | 2 | FA derivative | 1-C16:0-glycerol (2TMS) | 1 | 0.000707 | 0.000213 | 0.003053 | 0.001625 | 0.001302 | 0.006616 | 0.004368 | 0.000000 | 0.000230 | 0.002733 | 0.000285 | 0.000697 | 0.000287 | 0.002285 | 0.001125 | 0.000005 |
| 205 | e./l.stat | 188 | 2 | Saccharide | Ribose (Meox 4TMS) | 1 | 0.000249 | 0.000423 | 0.000095 | 0.000335 | 0.000336 | 0.000000 | 0.000653 | 0.000000 | 0.000067 | 0.000000 | 0.000140 | 0.000000 | 0.000000 | 0.000000 | 0.000236 | 0.000000 |
| 429 | l.stat | 381 | 2 | Saccharide | Maltose (Meox TMS) | 1 | 0.000089 | 0.000000 | 0.001595 | 0.000171 | 0.000379 | 0.003112 | 0.000254 | 0.000000 | 0.000026 | 0.000502 | 0.000020 | 0.000070 | 0.000050 | 0.000155 | 0.000074 | 0.000000 |
| 403 | exp | 351 | 2 | Saccharide | ? Galactosylglycerol (6TMS)/Glucopyranose (5TMS) | 3 | 0.000213 | 0.000021 | 0.002079 | 0.000241 | 0.000456 | 0.005191 | 0.000462 | 0.000002 | 0.000038 | 0.000952 | 0.000049 | 0.000269 | 0.000104 | 0.000298 | 0.000190 | 0.000000 |
| 281 | - | 235 | 2 | Saccharide | ? Glucose/Galactose (Meox 5TMS) | 3 | 0.016893 | 0.019588 | 0.053802 | 0.011080 | 0.013960 | 0.033822 | 0.012287 | 0.000000 | 0.000016 | 0.028379 | 0.003813 | 0.000000 | 0.000000 | 0.000034 | 0.008306 | 0.000000 |
| 180 | - | 162 | 2 | Saccharide | ?? Monosaccharide | 3 | 0.000417 | 0.001326 | 0.011196 | 0.001536 | 0.001975 | 0.000000 | 0.002418 | 0.000000 | 0.001507 | 0.000000 | 0.001074 | 0.000000 | 0.000000 | 0.000512 | 0.001789 | 0.000177 |
| 413 | e./l.stat | 367/8 | 2 | Saccharide | ?? Disaccharide | 3 | 0.000124 | 0.000017 | 0.000581 | 0.000242 | 0.000387 | 0.002652 | 0.000642 | 0.000002 | 0.000031 | 0.000905 | 0.000041 | 0.000228 | 0.000129 | 0.000462 | 0.000161 | 0.000000 |
| 341 | l.stat | 288 | 2 | Terpene | Phytol (1TMS) | 1 | 0.001412 | 0.000825 | 0.007134 | 0.003608 | 0.003183 | 0.006151 | 0.014572 | 0.000000 | 0.001335 | 0.008525 | 0.001575 | 0.003081 | 0.003726 | 0.009563 | 0.003543 | 0.000486 |
| M | l.stat | 151 | 2 | Unknown | - | 4 | 0.000058 | 0.000092 | 0.000307 | 0.000138 | 0.000118 | 0.000154 | 0.000173 | 0.000016 | 0.000031 | 0.000385 | 0.000032 | 0.000000 | 0.000120 | 0.000042 | 0.000071 | 0.000007 |

The full list is provided in a separate excel document (tab 'Table S8').

Supplementary Figure S1 Concentrations of nitrite (a), nitrate (b) and phosphate (c) in batch cultures of *Phaeocystis pouchetii* AJ01. Average ± SD concentrations within the algal cultures (n = 4) and one medium control (open circles) as measured by spectrophotometry (black filled circles) or ICPMS (grey filled circles) are shown. Red arrows indicate metabolite sampling time points.


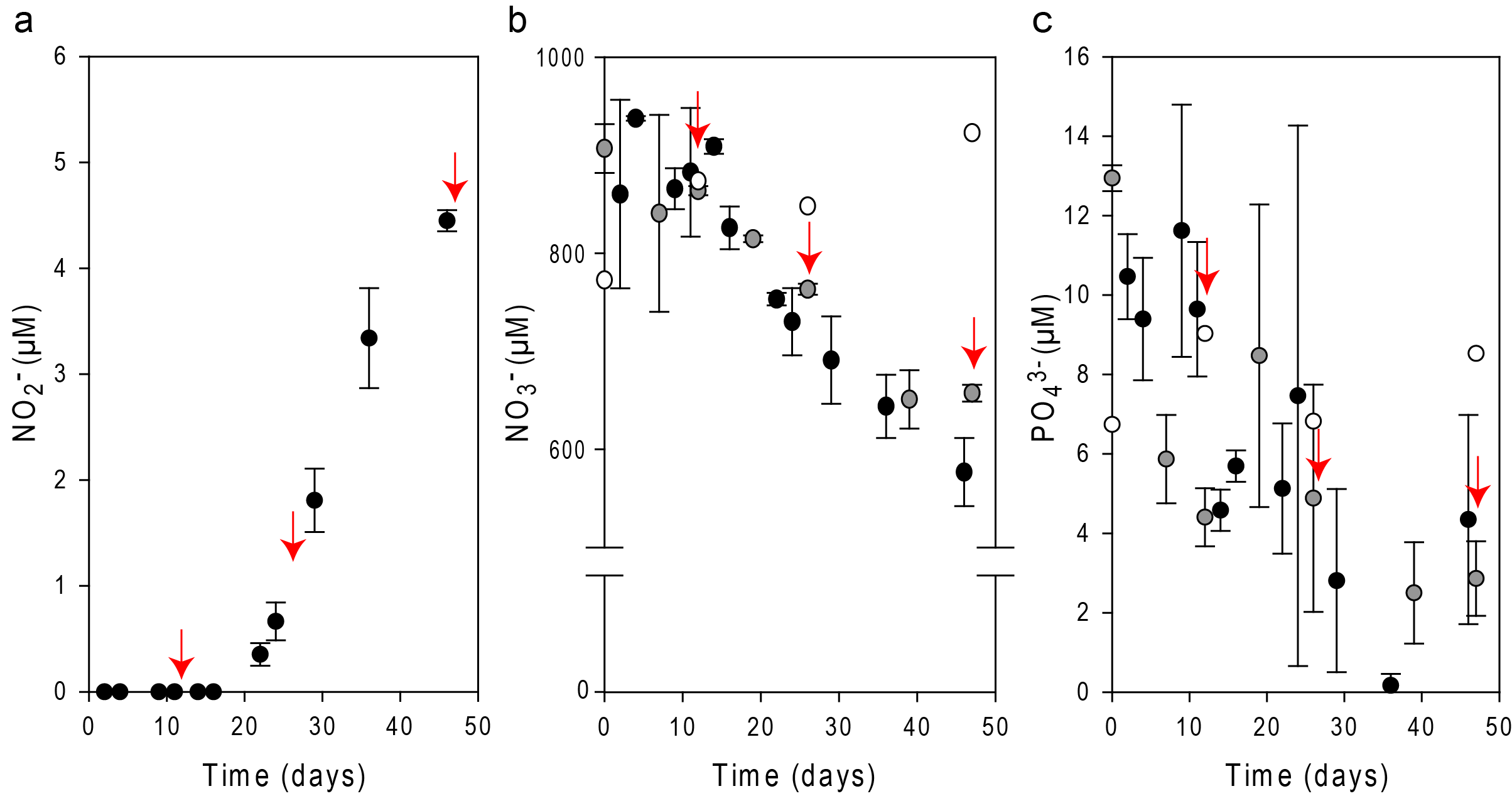


Supplementary Figure S2 Mass spectral and retention time characterization of a quinic acid derivative detected in the endometabolome of *Phaeocystis pouchetii* AJ01 correlating with the exponential and early stationary growth phase. a) Mass spectra of quinic acid reference standard and the unknown derivative. 1 - Characteristic fragments are listed as reported by Molnár-Perl et al. (1998). 2 - A typical fragment with *m*/*z* = 345 is shown as reported by Fuchs and Spiteller (1996). b) Extracted ion chromatograms (EICs) of *m*/*z* = 345 corresponding to the typical quinic acid fragment for the quinic acid reference standard and a representative algal extract (time scaled to retention time indices).


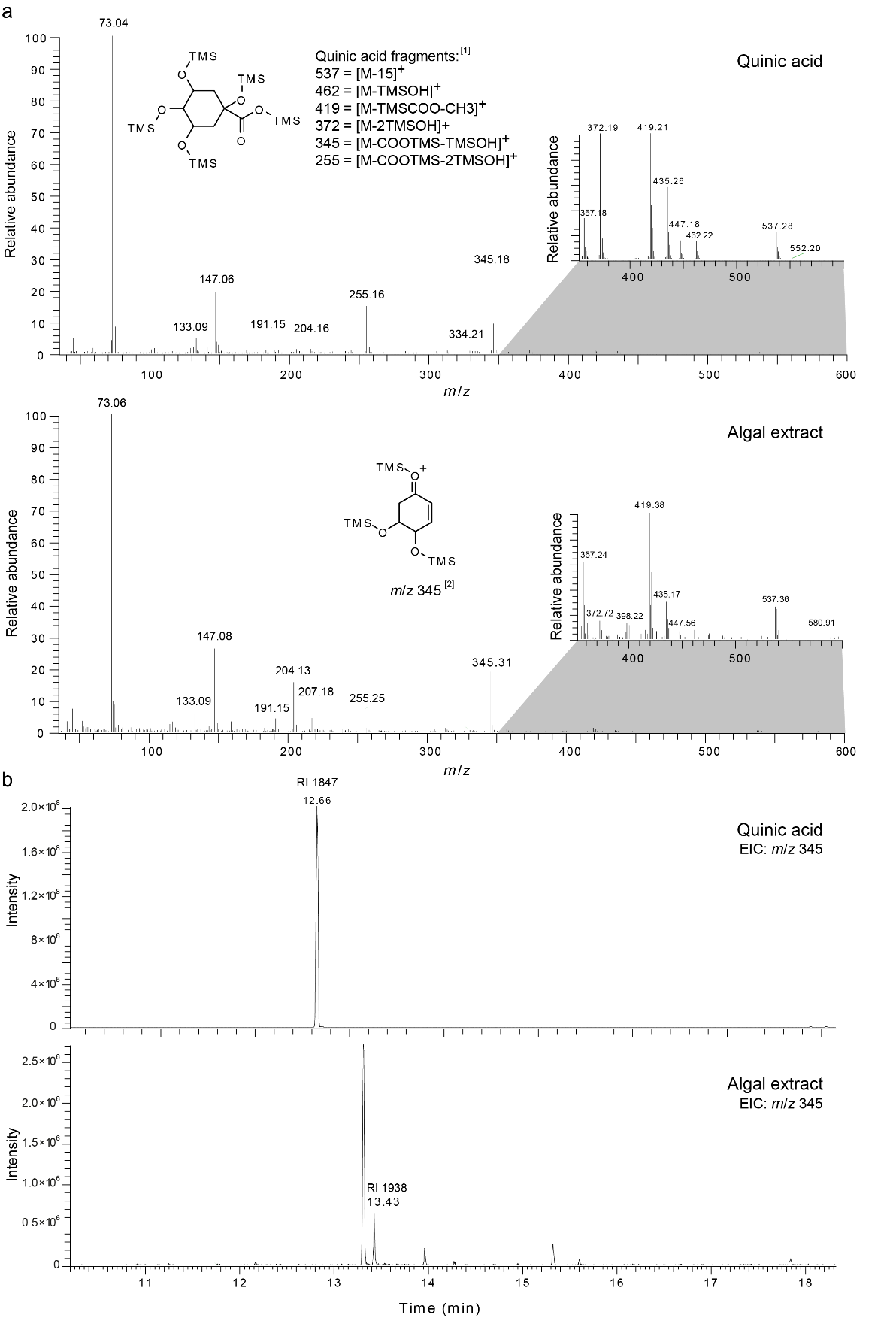


**Supplementary Figure S3** CTD profiles of PHAEONIGMA cruise stations sampled along a transect in the Barents Sea (Station 1-3) and in North Norwegian fjords (Station 4-6) including the following parameters: chlorophyll *a* fluorescence (green), temperature (blue), salinity (red) and density (black).


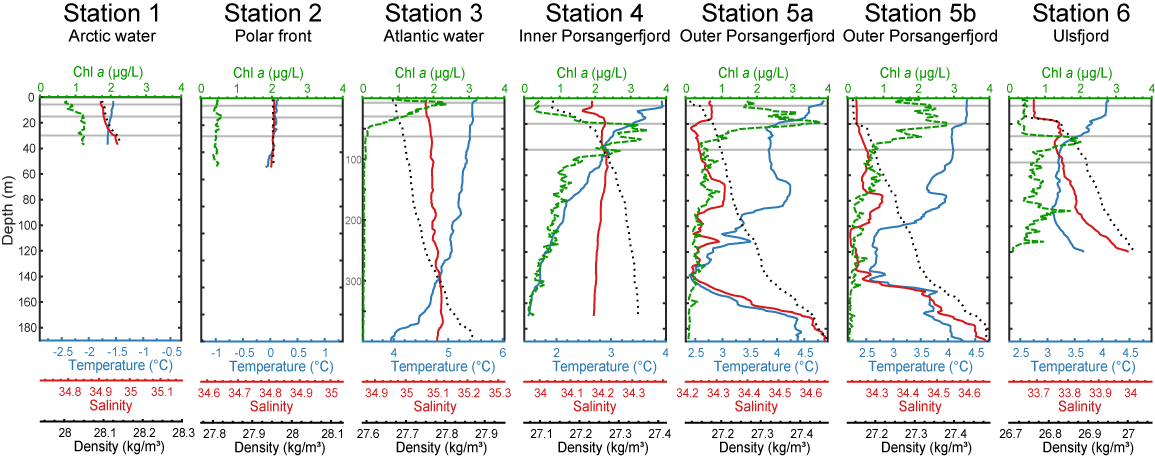


**Supplementary Figure S4** Typical FlowCam images of *Phaeocystis pouchetii* colonies obtained in the chlorophyll maxima of PHAEONIGMA cruise stations sampled along a transect in the Barents Sea (Station 1-3) and in North Norwegian fjords (Station 4-6). At Stations 1-2, no colonies were found. a-b) At Stations 3-4, colonies were small with firmly structured patches indicating early bloom stages. c) At Station 5a, colonies were larger and showed infestation with the pennate diatom *Pseudo*-*nitzschia* (black arrows) indicating a later bloom stage as described by Sazhin *et al.* (2007). d) At Station 5b, the degradation of the patch structure due to formation of flagellated cells is visible (white arrow) in addition to an infestation with *Pseudo*-*nitzschia* (black arrow). e) At Station 6, colonies were dominated by the formation of flagellated cells (white arrows) indicating the latest stage of a *Phaeocystis* bloom of all stations.


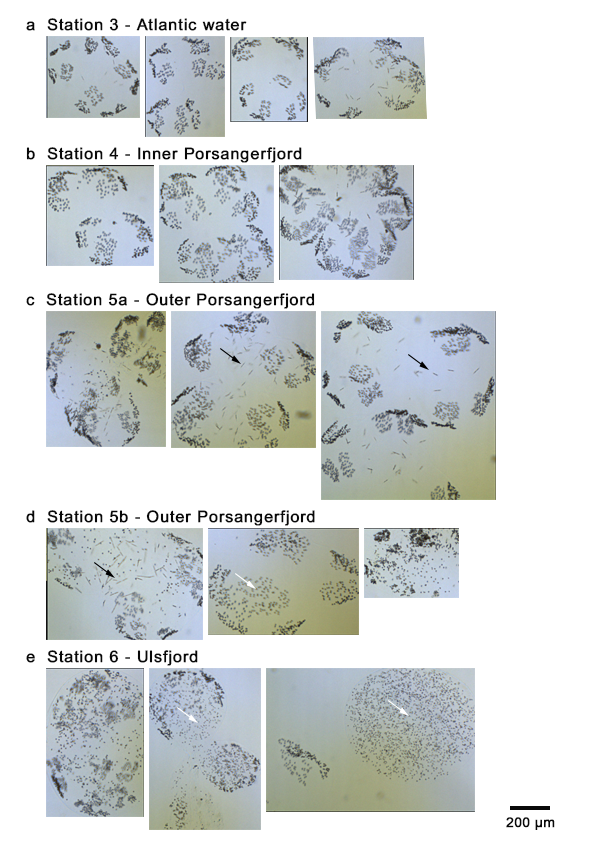


Supplementary Figure S5 Abundance of *P. pouchetii* endometabolites at the stations sampled during the PHAEONIGMA cruise along a transect in the Barents Sea (Station 1-3) and in North Norwegian fjords (Station 4-6). Heat map shows endometabolites (peak area/peak sum) averaged per cruise station and sorted by hierarchical cluster analysis using Pearson distance measure and Ward cluster algorithm. Metabolite identifier of field dataset indicated on the right.


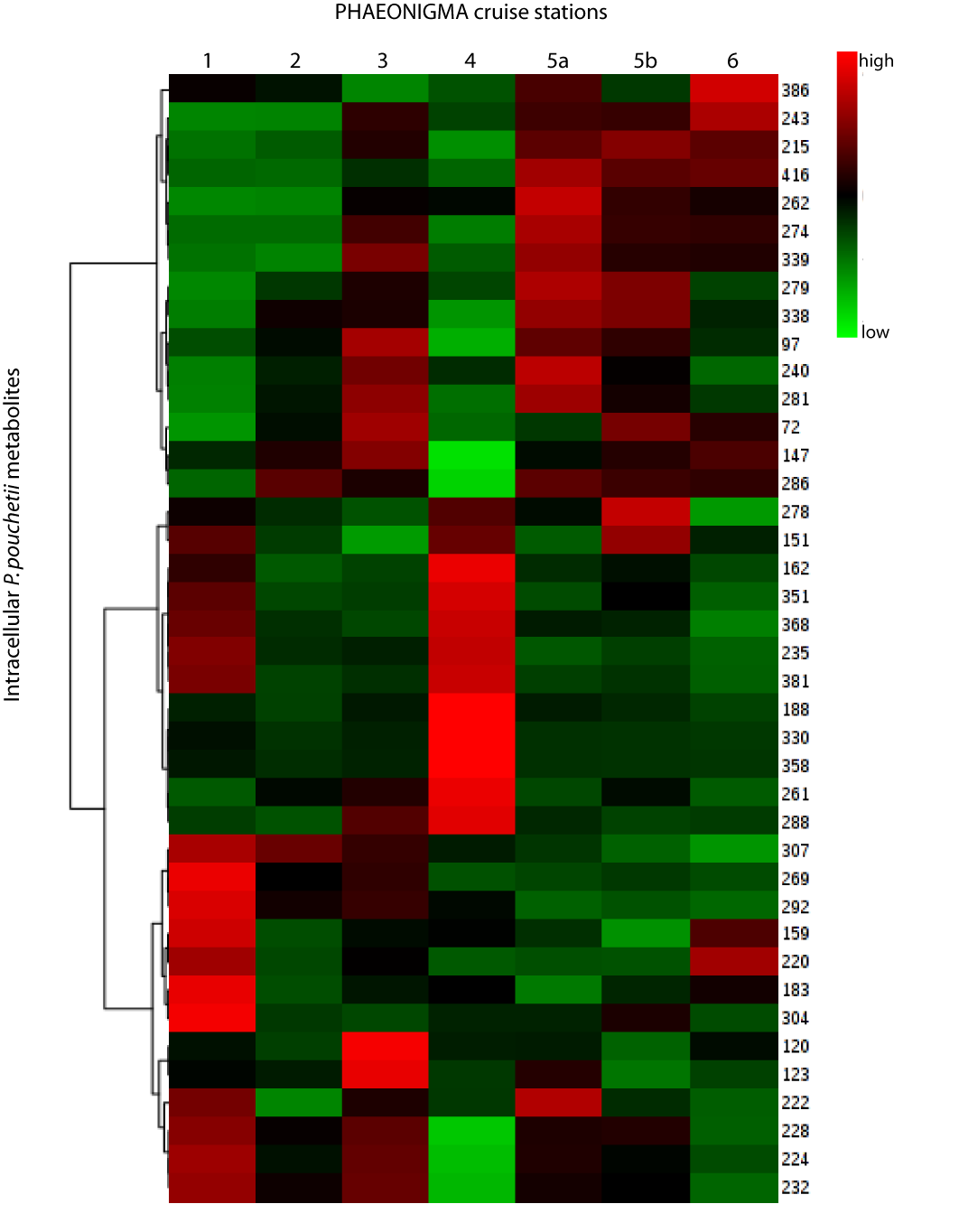


**Method references:**

Fuchs, C. & Spiteller, G. Rapid and easy identification of isomers of coumaroyl‐ and caffeoyl‐D‐quinic acid by gas chromatography-mass spectrometry. *J. Mass Spectrom.* **31**, 602-608 (1996).

Molnár-Perl, I., Vasanits, A. & Horváth, L. Simultaneous GC-MS quantitation of phosphoric, aliphatic and aromatic carboxylic acids, proline and hydroxymethylfurfurol as their trimethylsilyl derivatives: In model solutions II. *Chromatographia* **48**, 111-119 (1998).

Sazhin A.F., Artigas, L.F., Nejstgaard, J.C. & Frischer, M.E. The colonization of two *Phaeocystis* species (Prymnesiophyceae) by pennate diatoms and other protists: a significant contribution to colony biomass. *Biogeochemistry* **83**, 137-145 (2007).

Sumner, L.W. *et al.* Proposed minimum reporting standards for chemical analysis. *Metabolomics* **3**, 211-221 (2007).
